# Egyptian Blue into Carboxymetylcellulose: New Dual-Emissive Solid-State Luminescent Films

**DOI:** 10.3390/molecules30112359

**Published:** 2025-05-28

**Authors:** Mariana Coimbra, Francesco Fagnani, Gisele Peres, Paulo Ribeiro-Claro, Juan Carlos Otero, Daniele Marinotto, Dominique Roberto, Mariela Nolasco

**Affiliations:** 1 CICECO—Instituto de Materiais de Aveiro, Departamento de Química, Universidade de Aveiro, P-3810-193 Aveiro, Portugal; marianacoimbra@live.com.pt (M.C.);; 2 Dipartimento di Chimica, Università degli Studi di Milano, UdR-INSTM, Via C. Golgi 19, I-20133 Milan, Italy; 3 Engenharia de Alimentos, Federal University of Fronteira Sul, Campus Laranjeiras do Sul, Laranjeiras do Sul 85301-970, PR, Brazil; gisele.peres@ua.pt; 4 Department of Physical Chemistry, Faculty of Sciences, University of Málaga, E-29071 Málaga, Spain; jc_otero@uma.es; 5 Istituto di Scienze e Tecnologie Chimiche (SCITEC) “Giulio Natta”, Consiglio Nazionale delle Ricerche (CNR), Via C. Golgi 19, I-20133 Milan, Italy; daniele.marinotto@cnr.it

**Keywords:** sodium carboxymethylcellulose, Egyptian blue, clustering-triggered emission, NIR luminescence, citric acid

## Abstract

The development and characterization of a sustainable carboxymethylcellulose (CMC)-based material hosting Egyptian blue (EB) as a luminophore with emission in both the visible and NIR regions is herein presented and discussed, demonstrating its potential to be applied in a variety of applications, such as bioimaging, sensing, and security marking. Solution casting was used to synthesize the films, with citric acid (CA) as a crosslinking agent. Fully characterization was performed using attenuated total reflection (ATR) and coherent anti-Stokes Raman scattering (CARS) spectroscopy, zeta potential, UV–Vis, and photoluminescence (PL) spectroscopy, and thermal analysis techniques, such as thermogravimetric analysis (TGA) and differential scanning calorimetry (DSC). The results confirm the effective crosslinking of CMC with CA within CMC–EB–CA films with 1.5 and 3% of EB. The introduction of EB retained its usual NIR emission with λ_em_ max = ~950 nm reaching quantum yield values in the range of 11.2–12.8% while also enabling a stable dispersion within the CMC matrix, as confirmed using CARS imaging and zeta potential. Additionally, the CMC films exhibited the characteristic clustering-triggered emission (CTE) in the blue region at 430 nm with a slight increase in luminescence quantum yield (Φ) from 5.8 to 6.1% after crosslinking with citric acid.

## 1. Introduction

Natural polymer-based fluorescent materials showing the advantages of biodegradability, biocompatibility, eco-friendliness, and sustainability have emerged as a key focus of functional polymer materials in the cross-cutting frontier fields of life science and chemistry [1].

In contrast to traditional organic luminophores with double bonds and aromatic rings, the unique luminescence behavior of non-traditional luminescent materials without π-units has been discovered [2,3,4,5,6,7]. Instead of aromatics, non-traditional luminophores are characterized by a nonconjugated structure with saturated C–N, C–O, or C–C bonds or/and electron-rich heteroatoms. Today, the clustering-triggered emission (CTE) mechanism can give a good explanation for the bright emission of non-traditional luminophores in the solid state and at high concentrations [2,3,4,5,6,7]. This happens when electron-rich moieties, without significant conjugation groups, produce electron cloud overlap by means of a clustering space conjugation behavior.

Some natural polysaccharides, like chitosan, starch, cellulose, and sodium alginate, are characterized by a blue photoluminescence due to the CTE mechanism [8,9,10,11,12,13,14]. All of them have O atoms with lone electron pairs, in addition to H and C atoms. Thus, the clusterization of O atoms with efficient O···O interactions enable electron overlapping [6].

Over the years, researchers have adopted various methods to synthesize long-lived and multicolored natural polysaccharide-based luminescent materials [15,16,17,18,19]. Among these, cellulose stands out and, in fact, has been developed into various forms of cellulose-based fluorescent materials, such as solution, film, fiber, coating, hydrogel, aerogel, etc. [1]. Regrettably, the blue fluorescence of cellulose-based materials—attributed to their intrinsic n→π* and σ→σ* transitions—is low and has not yet reached the level of required practical applications [1] despite the efforts. Therefore, it is of great significance to optimize the luminescence of cellulose to meet practical application needs.

In 2018, Du et al. [9] demonstrated that carboxymethylcellulose (CMC) also presented blue fluorescence from the CTE mechanism due to the clustering of oxygen atoms and carboxyl groups (-COOH). Additionally, with multicarbon functional groups on its surface, like hydroxide (-OH) and carboxylate (-COO^−^), CMC can be modified by crosslinking through an esterification-based mechanism using citric acid (CA) [20]. The newly added -COOH groups from citric acid and C=O groups from the ester moieties in the cellulose backbone may somehow enhance the blue fluorescence. Citric acid (CA) has demonstrated efficacy as a crosslinking agent for carboxymethylcellulose (CMC), facilitating the creation of a three-dimensional network that promotes uniform polymer chain distribution while reducing structural imperfections. This crosslinking process yields polymer films that are sustainable, biodegradable, biocompatible, environmentally friendly, non-toxic, and cost-effective, while also exhibiting uniformity and structural cohesion—qualities essential for a wide range of applications [20,21,22,23].

The development of CMC-based materials with multichannel fluorescence capability is a research hotspot, and the incorporation of a fluorophore emitting in the near infrared (NIR) in the CMC matrix would expand the number of applications. The broad excitation band in the visible part of the spectrum of Egyptian blue (EB) and its emission in the NIR region makes it a promising candidate.

EB is an ancient synthetic pigment with the same structure as the mineral cuprorivaite (CaCuSi_4_O_10_) [24,25]. It is strongly luminescent in the NIR region (800–1100 nm) with an excited state lifetime of 107–159 μs and a λ_em_ max of ~950 nm when excited with red light (λ_ex_ = 600–630 nm) [26,27]. Its luminescence has been attributed to d-d transitions, sensitive to the crystal field strength, taking into account that the Cu^2+^ ion in D_4h_ is the only luminescent center [28].

Because of its such peculiar properties, many interesting applications of EB in different fields have been developed, such as in optical chemosensors [29], optical sensing materials [30], fingerprint dusting powder [31], security inks [32], Li-ion batteries [33], bioimaging [34], and luminescent solar concentrators [35,36].

To the best of our knowledge, the development of a sustainable CMC-based material hosting EB as a luminophore with emissions in both the visible and NIR regions has still not been reported and is herein presented. Due to the excellent film forming property of CMC, films crosslinked with CA are usually prepared using the solution casting method [37]. The choice of the deposition methods plays a critical role in determining the electronic and optoelectronic properties of the material because it has an influence on the resulting microstructure [38]. However, methods, like spin coating, are known for fast solvent removal from surfaces. This could restrict CA diffusion within the CMC matrix before the thermal cure, thus hindering the formation of an integrated three-dimensional crosslinked network. Thus, in this work, the CMC–EB–CA films were prepared via solution casting.

Fully characterization was performed by attenuated total reflection (ATR), zeta potential (ZP), UV–Vis, and photoluminescence (PL) spectroscopy. Additional information about surface morphology was provided by coherent anti-Stokes Raman scattering spectroscopy (CARS) and atomic force microscopy (AFM). Thermal properties were also analyzed by means of thermogravimetric analysis (TGA) and differential scanning calorimetry (DSC) techniques.

## 2. Results and Discussion

### 2.1. ATR Spectroscopy

The carboxymethylcellulose films were crosslinked by means of an esterification-based mechanism with citric acid [20]. This mechanism was reported in various studies in which a carboxylic acid was used to crosslink polysaccharides, such as cellulose or starch derivatives [39,40,41]. The crosslinking mechanism occurs by heating the films, producing the conversion of CA into citric anhydride [42], which reacts with the hydroxyl groups of the polysaccharide followed by dehydration of the CA carboxylic groups and reaction with other hydroxyl groups leading to the crosslinking between the polymer chains [39,43,44]. In our study, the CMC samples are abbreviated from F0 to F4 according to EB and/or CA content (F0: pure CMC; F1: with 10% *w*/*w* of CA; F2: with 1.5% *w*/*w* EB; F3: with 1.5% *w*/*w* EB and 10% *w*/*w* CA; F4: with 3% *w*/*w* EB and 10% *w*/*w* CA; see Section 3. Materials and Methods).

**Figure 1:**
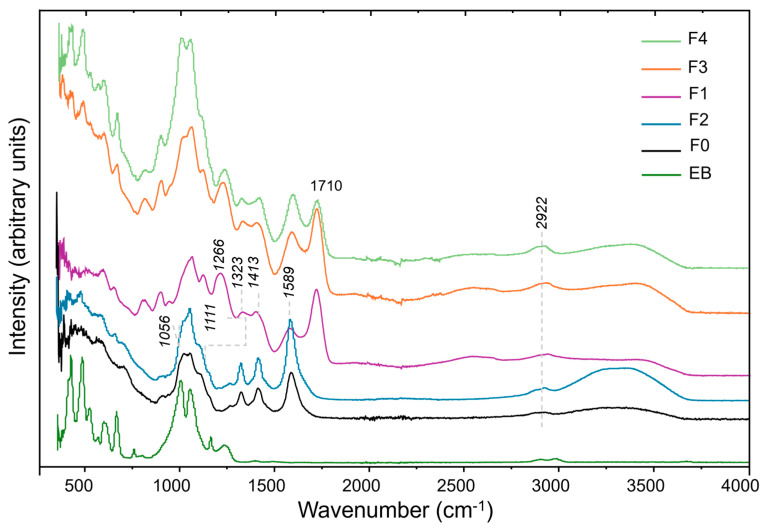
ATR spectra of Egyptian blue (EB) powder and films of carboxymethylcellulose (CMC) (F0), citric acid (CA)-crosslinked (CMC-CA) (F1), non-crosslinked (CMC--EB) (F2), and crosslinked (CMC–EB-CA) (F3, F4).

The success of the crosslinking process with CA was confirmed by ATR (Figure 1). All of the F0–F4 film spectra display the main absorption bands of the CMC backbone, as shown in Figure 1. Additionally, the comparison with CA itself is provided in Figure S1 of the ESI. The band at 2922 cm^−1^ is assigned to the stretching of C–H bonds, the two bands at 1589 cm^−1^ and 1413 cm^−1^ are assigned to the symmetric and antisymmetric deformation of CMC’s carboxylate anion (COO^−^), respectively, the band at 1323 cm^−1^ derives from the deformation of the group CH–O–CH_2_, and the band at 1266 cm^−1^ arises from the stretching of the C–O bonds. The bands at 1111 cm^−1^ and 1056 cm^−1^ are characteristic of the deformation of carboxymethyl C–O and C–O–C substituent groups [45]. The appearance of the band at 1710 cm^−1^, attributed to the ester carbonyl group, confirms the formation of ester linkages following the crosslinking reaction as already observed in the case of other cellulosic films [44]. As this band is not detected in non-crosslinked F0 and F2 films (Figure 1), it can be used as a probe to confirm crosslinking. Additionally, the ATR spectra of CMC–EB-CA films lack the characteristic doublet of the citric acid carboxyl (-COOH) stretching modes at 1720 and 1751 cm^−1^ (CA itself at Figure S1 of the ESI), confirming that free citric acid is not present.

The bands from EB, namely the strong bands observed at ca. 421 and 481 cm^−1^, assigned, respectively, to the bending modes of δ SiO_2_ and δ SiOSi [22], are also observed in the F3 and F4 film spectra, thus confirming the success of the incorporation of the EB into the CMC matrix.

### 2.2. Thermal Analysis

**Figure 2:**
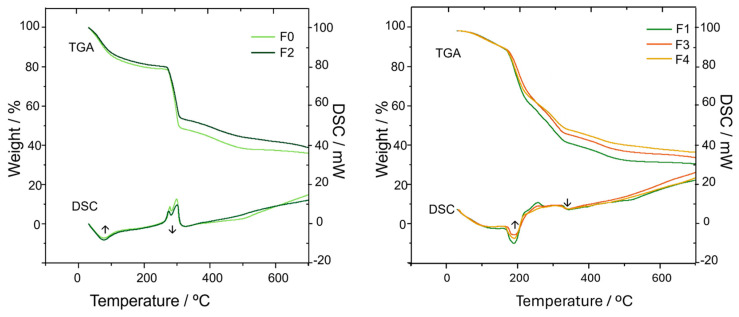
DSC and TGA analysis of non-crosslinked carboxymethylcellulose (CMC) (F0) and (CMC--EB) (Egyptian blue) (F2) films (A), and citric acid (CA)-crosslinked (CMC-CA) (F1) and (CMC–EB-CA) (F3, F4) films. The symbols ↑ and ↓ on the DSC curves indicate the directions of the endothermic and exothermic peaks, respectively.

Differential scanning calorimetry (DSC) and thermogravimetric analysis (TGA) were also performed (Figure 2). The thermograms clearly show some significant differences between them that also evidence the success of the crosslinking process.

Regarding the TGA analysis, it is observable that the degradation of the non-crosslinked CMC films, F0 and F2, is completed in three steps, which are also described in the literature [42,46]. In the first degradation step (30–115 °C), due to the loss of absorbed water, both F0 and F2 exhibit a weight loss of 15%. The second degradation step occurs when a temperature of 274 °C is reached, and it is attributed to the degradation of the side chain of the CMC polymer and the loss of CO_2_. This is the most prominent degradation step, consisting of a weight loss of 30% and 25% for F0 and F2, respectively.

The last degradation step begins at 310 °C and is ascribed to the degradation of the main chain of the polymer. Here, the F0 presents a higher weight loss than the film with the EB incorporated (F2). This agrees with the fact that EB is a non-volatile compound, hence contributing to the higher remaining mass of the films where it is incorporated.

In the crosslinked F1, F3, and F4 films, the loss of water is followed by several less defined degradation steps, starting at ca. 70 °C.

Concerning the DSC analysis, the presence of endothermic peaks at ca. 80 °C, in both the non-crosslinked (F0, F2) and crosslinked films (F1, F3, F3), is in agreement with the literature that assigns the peaks to the release of water molecules present in the structure [40,47].The higher temperature in the crosslinked films points out that the crosslinking makes the release of water molecules more difficult. The endothermic peak at 190 °C is clearly a distinctive feature of the crosslinked films. Nevertheless, the literature is quite ambiguous concerning the origin of this endothermic event [40,46,48] being the degradation of citric acid a possible explanation. Additionally, the non-crosslinked F0 and F2 films display two well-defined exothermic peaks at 280–300 °C that correspond to the formation and release of CO_2_ discussed above in the TGA analysis. Regarding the crosslinked F1, F3, and F4 films, these exothermic peaks occur across a wider range of temperatures, 210–320 °C, which might be due to the lack of free COO^−^ groups to release CO_2_, since these CMC groups are involved in the crosslinking with citric acid.

The presence of EB accounts for some visible changes in the DSC curves. For instance, the exothermic transition profile observed at 210–320 °C in the crosslinked films is undoubtedly affected by the increase in the amount of EB. This goes beyond what is expected due to the presence of an inert species with a different heat capacity, which suggests an interaction between the EB and the CMC matrix in the thermal degradation process.

### 2.3. Zeta Potential

**Figure 3:**
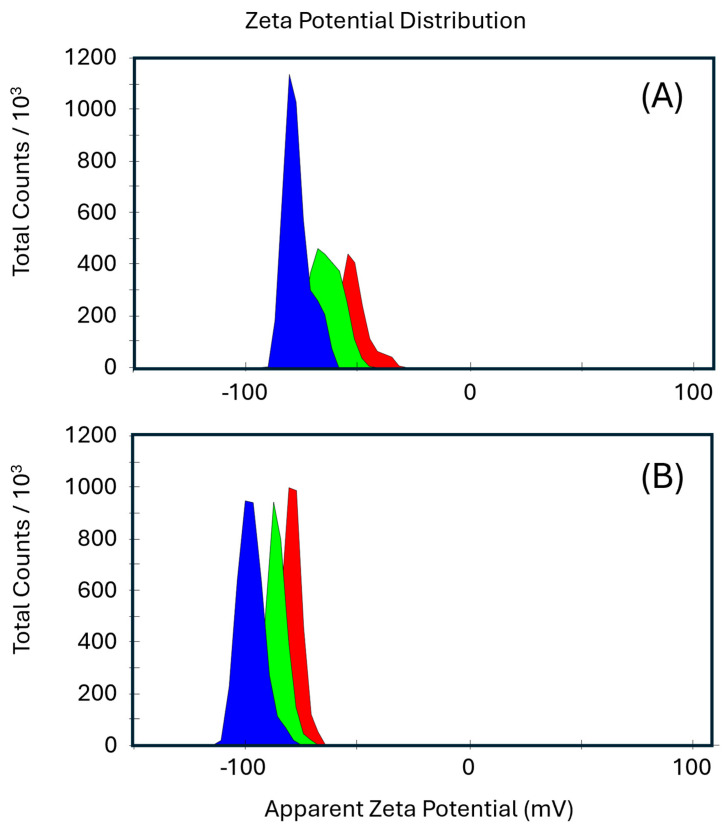
Zeta potential distribution graphs for carboxymethylcellulose (CMC) (**A**) and CMC–EB (Egyptian blue) (**B**) solutions.

The analysis of the electrokinetic properties and stability revealed that the apparent zeta potential (ZP) is a key parameter for assessing the surface charge of particles in suspension, directly impacting the colloidal stability of the matrix. Studies have shown that modifications to CMC can substantially alter its ZP, thereby affecting its ability to interact with other components [49,50]. In this context, the ZP distribution graphs (Figure 3) indicate an improvement in the uniformity of the particle population upon the addition of EB (CMC–EB). In contrast, CMC exhibits a broader and less homogeneous distribution. This suggests that the presence of EB facilitates a more even redistribution of surface charges, enhancing electrostatic stabilization and improving colloidal dispersion. These findings were further corroborated by electrophoretic mobility and ZP measurements.

**Figure 4:**
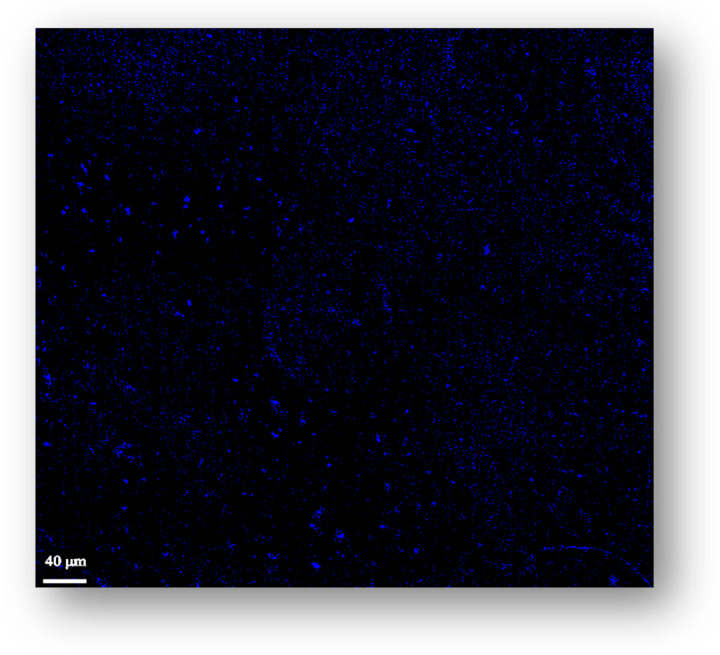
Coherent anti-Stokes Raman scattering (CARS) image of F3 film depicting the blue Egyptian blue (EB) particles dispersed in the citric acid (CA)-crosslinked carboxymethylcellulose (CMC–CA) matrix.

CMC exhibited a ZP of −63.7 mV, indicating good colloidal stability due to strong electrostatic repulsion among negatively charged particles. Upon complexation with EB, the ZP significantly decreased to −87.9 mV, suggesting an increase in the net negative charge. This result implies that EB interacts electrostatically with the carboxylate groups of CMC, reinforcing the stability of the colloidal system. Additionally, the increased negative charge could indicate that EB contributes extra anionic sites or promotes further dissociation of carboxylate groups, intensifying electrostatic repulsion within the system (Figure 4).

The electrophoretic mobility followed a similar trend, shifting from −4.990 µm·cm/V·s (CMC) to −6.894 µm·cm/V·s (CMC--EB). This increase in mobility further supports the hypothesis of enhanced charge density and stronger repulsion forces within the complexed system, reinforcing the idea that EB alters the local electrostatic environment. Moreover, the reported results represent the mean values of triplicate measurements, ensuring statistical reliability.

The conductivity of the CMC solution was 3.50 mS/cm, whereas the incorporation of EB reduced it to 2.28 mS/cm. This reduction suggests that dye molecules are participating in ion exchange interactions, potentially binding to available carboxylate sites, thereby decreasing the number of free ionic species in solution. Furthermore, the possible aggregation induced by EB could reduce the mobility of charge carriers, contributing to the observed decrease in conductivity.

In addition to the changes in ZP, the pH values of the solutions provide further insight into the interaction between CMC and EB. The pH of the CMC solution was 7.22, which is typical for a neutral to slightly basic environment where CMC is stable. However, the pH of the CMC + EB solution increased to 7.92, suggesting that EB may have a buffering effect, slightly raising the pH of the solution. This increase in pH can be attributed to electrostatic interactions between EB and the carboxylate groups of CMC, which may alter the charge distribution and influence hydrogen ion availability. More specifically, EB could neutralize acidic sites on CMC, reducing the overall concentration of free protons in solution. Additionally, its ionic nature might facilitate the release of bound metal ions or engage in specific binding with functional groups in CMC, further contributing to the observed shift in pH and zeta potential. The buffering capacity of EB appears to contribute to system stability by mitigating pH fluctuations, thereby reinforcing the electrostatic balance within the CMC matrix.

Thus, the rise in pH, alongside the enhanced colloidal stability and ZP modifications, confirms the establishment of strong interactions between EB and the CMC structure. These findings suggest that EB not only modifies the electrostatic properties of the polymer but also contributes to maintaining system stability, likely through charge redistribution and pH regulation.

### 2.4. Microscopy Imaging

Coherent anti-Stokes Raman scattering (CARS) is a nonlinear Raman spectroscopy technique that provides unique conditions for microscopy imaging. CARS microscopy was herein used to generate spatially well-defined images of the crosslinked film, i.e., CMC–EB-CA (F3), so that the dispersion of the EB in the matrix could be assessed.

To acquire a significant CARS signal, the beat frequency must correspond to the Raman active vibrational mode of the sample, in this case EB, since we wanted to map its distribution within the film. Considering the Raman spectrum of EB [24], one of the most characteristic and intense vibrational modes occurs at ca. 430 cm^−1^. As no vibrational mode is observed in this region in the Raman spectra of CMC [51] and CA [52], the beat frequency has been set to the wavenumber of 430 cm^−1^.

The selected vibrational mode of EB at 430 cm^−1^ enters in resonance with the beat frequency—the difference between the energy of the two laser beams ϖ_pump_ and ϖ_Stokes_—and the spots where a strong anti-Stokes signal at (ϖ_pump_ − ϖ_Stokes_) + ϖ_pump_ are represented in a false color in the microscope image, making it possible to observe the EB particles, in this case also in bright blue, dispersed in the crosslinked CMC matrix, as shown in Figure 4. As all the other vibrational modes are off-resonance, no signal is produced by them.

The obtained CARS image clearly reveals a predominantly uniform spatial distribution of EB within the crosslinked CMC–CA matrix. Moreover, the image enables the measurement of the average size of EB particles, which range from 1 to 4 µm, as indicated by the provided scale.

The surface morphology of the F1 film was analyzed using atomic force microscopy (AFM). Topographic images obtained at different scan areas (Figure S2 of the ESI: 5 µm, 1 µm, and 500 nm) revealed a smooth and continuous surface, free from visible discontinuities or phase separation. At smaller scales, the presence of well-distributed nanostructures and the absence of morphological defects further supports the conclusion that efficient and uniform crosslinking was achieved throughout the CMC matrix.

### 2.5. UV–Vis and PL Spectroscopy

In the UV–Vis spectra of F0 and F1 (Figure S3 of the ESI), a peak maximum at 225 nm and a broad absorption shoulder in the range of 250–350 nm—attributed to n-π* electronic transitions in the carboxyl (-COO^−^) and carbonyl (C=O) groups of CMC—are observed [9].

After the crosslinking reaction, the CMC optical behaviour slightly changed, with the shoulder peaking at ca. 350 nm being enhanced when compared with F0. This may be related to the electronic transitions of the ester groups created after crosslinking reaction of CMC with CA.

**Figure 5:**
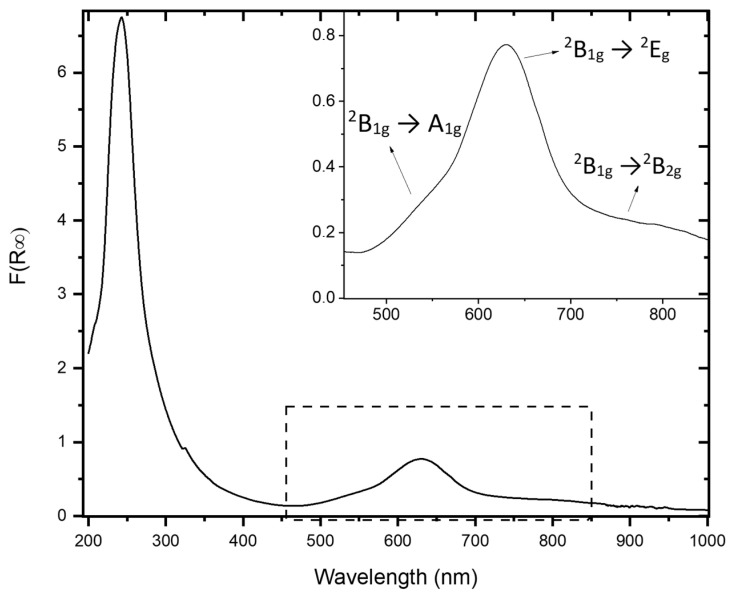
Kubelka–Munk function, F(R_∞_), of the Egyptian blue (EB) powder. Inset: magnification of the spectrum in the visible range.

The optical absorption spectrum of the EB powder was recorded in diffuse reflectance mode, and the Kubelka–Munk function is shown in Figure 5. The absorption bands at about 784, 629, and 549 nm, corresponding to three different electronic transitions, namely ^2^B_1g_ →^2^B_2g_, ^2^B_1g_ → ^2^E_g_, and ^2^B_1g_ → A_1g_, respectively, are attributable to Cu^2+^ ions under D_4h_ symmetry [9,27,28], while the absorption band at 242 nm is related to the transition from the valence band to the conduction band [26].

**Figure 6:**
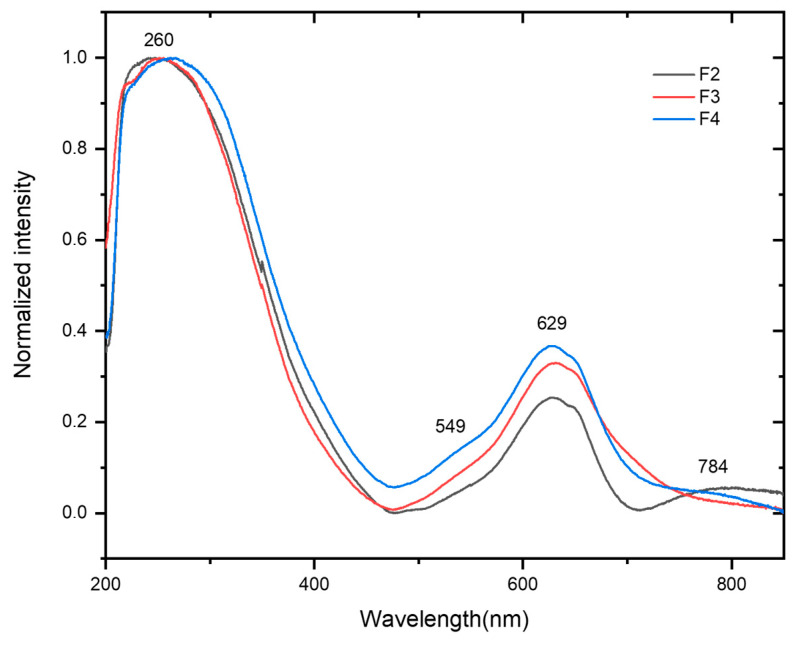
UV–Vis absorption spectra of non-crosslinked carboxymethylcellulose (CMC–EB) (Egyptian blue) (F2) and citric acid (CA)-crosslinked (CMC–EB-CA) (F3 and F4) films.

When EB is incorporated into the CMC (F2) and crosslinked CMC (F3, F4) matrix, it is observed that the peak maximum of CMC tends to shift toward longer wavelengths, from 225 nm (Figure S3 of the ESI) to 260 nm (Figure 6), which is characteristic of changes in the degree of crystallinity of CMC [53]. Concerning the EB optical properties, F2, F3, and F4 all kept the EB absorption bands practically unchanged.

Through naked-eye observation of non-crosslinked and crosslinked CMC films (F0 and F1)—under 365 nm UV irradiation—a blueish emission was quite evident, and the concentration of light emission on the edges of the films was outstanding (shown in Figure S4 of the ESI) evidencing the noticeable waveguide property of CMC films [54,55]. According to Lin-Lin Du et al. [9], the absorption and emission phenomena of CMC films can be explained by the clustering-triggered emission (CTE) mechanism, where the clustering of hydroxyl and carbonyl moieties results in intra- and inter-molecular interactions among them. These interactions create extended electron delocalization which can result in excitation and give rise to radiative emission.

**Figure 7:**
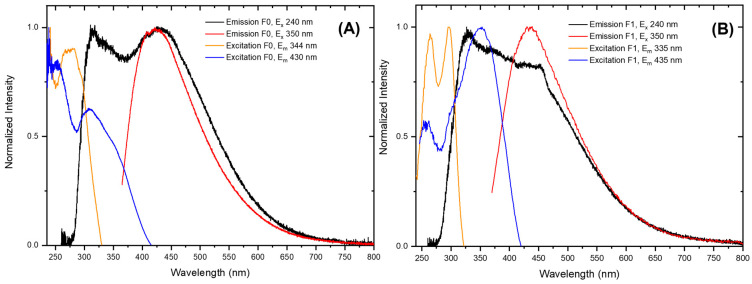
Normalized emission (**A**) and excitation (**B**) spectra of carboxymethylcellulose (CMC) (F0) and citric acid (CA)-crosslinked (CMC–CA) (F1) films with different wavelengths.

Figure 7 shows that both non-crosslinked (F0) and crosslinked (F1) CMC films have emission spectra that depend on the excitation wavelength. Such behaviour has been previously reported for other nonconventional luminophores with a CTE mechanism [6,8,9,10,11,12,13,14] and is related to the presence of the heterogeneous clusters constituting the film. Since different clusters can have different energy levels, it is possible to selectively modify the emission spectrum by choosing an appropriate excitation wavelength. Based on the emission data reported below, we hypothesize the existence of at least two different types of clusters. To better highlight the main photophysical properties of the F0 and F1 films, we added a simplified Jablonski diagram in Figures S5 and S6 of the ESI, respectively.

A broad emission band is observed when the sample is excited at high energy (240 nm) with maxima at around 316 (average lifetime τ_av_ of 1.10 ± 0.03 ns) and 330 nm (τ_av_ = 1.64 ± 0.09 ns) for the F0 and F1 films, respectively. Although in a subtle way, the newly added -COOH groups from citric acid and C=O groups from the ester moieties in the cellulose backbone somehow enhance the luminescence quantum yield. In fact, intense emission peaks at 430 (τ_av_ = 5.87 ± 0.09 ns) and 435 nm (τ_av_ = 3.87 ± 0.10 ns), with absolute luminescence quantum yields (Φ) of 5.8 and 6.1%, are obtained when both F0 and F1 films are excited at 350 nm, respectively. Luminescence time-resolved measurements of the excited states and related fits are reported in Table S1 and Figures S7–S10 of the ESI.

**Figure 8:**
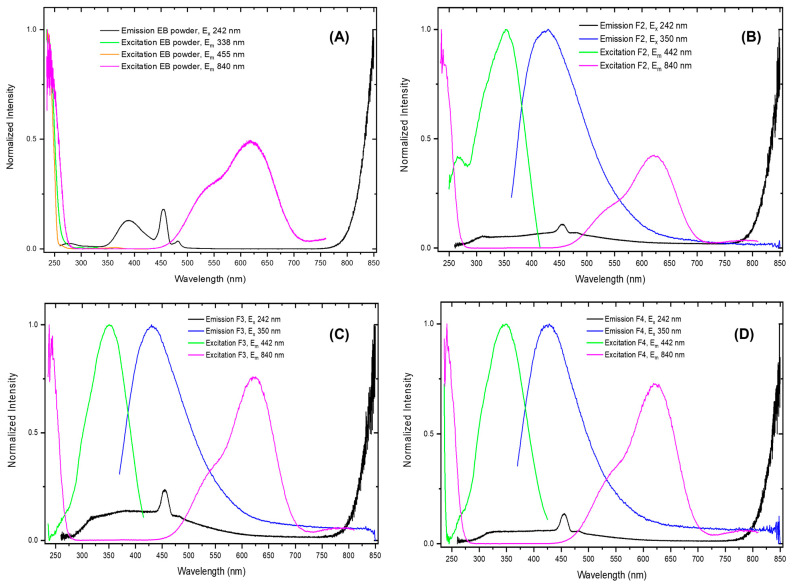
Normalized emission and excitation spectra of Egyptian blue (EB) powder (**A**) and of F2 (**B**), F3 (**C**), and F4 (**D**) films.

**Figure 9:**
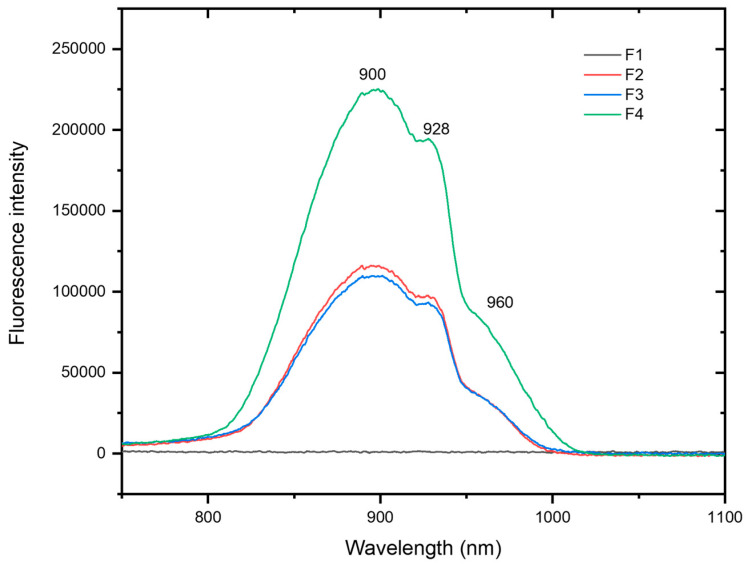
Fluorescence spectra of F1–F4 films in the NIR region, with λ_exc_ = 630 nm.

Emission and excitation spectra in the UV–visible range of EB powder and of the F2, F3, and F4 films are shown in Figure 8, with the relative simplified Jablonski diagrams in Figures S11–S13 of the ESI. Upon excitation at 350 nm, an emission peak at about 430 nm is observed for the F2–F4 films, while no emission is detected for the EB. The absolute luminescence quantum yield of this emission in the F2, F3, and F4 films is 1.3, 2.0, and 1.4%, with an average lifetime of 4.79 ± 0.06 ns, 4.43 ± 0.06 ns, and 4.59 ± 0.06 ns (Figures S14–S16 of the ESI), respectively. These values are like those already observed for the analogous emission in the F0 and F1 CMC films (see Table S1), with only a slight quenching in the quantum yield, probably due to the absorption of the EB luminophore. Therefore, the emission observed at 430 nm can be attributed to the non-crosslinked (F2) and crosslinked CMC matrix (F3 and F4), respectively. Upon excitation at 242 nm, the emission spectra of the F2, F3, and F4 films in the UV–visible range display an initial broad band extending from 300 to 700 nm, with a peak centered at 455 nm, and a second band which starts to rise from 750 nm (see Figure 9 for a complete description of the NIR emission spectrum).

The broad emission band is quite like those of F0 and F1 matrices when excited at the same wavelength (see Figure 8); however, it is possible to recognize additional peaks (390, 455, and 481 nm) which are related to the emission of the EB luminophore, as can be observed in Figure S17 of the ESI. According to the literature [26,56,57] these additional peaks are assigned to the recombination of electron–hole pairs (excitons) either self-trapped or trapped by defects in the EB, which are maintained after incorporation into the CMC matrix. In particular, the observed emission bands at 390 and 481 nm are assigned to trapped exciton emission—where the hole is only trapped at Cu^2+^ sites, giving Cu^3+^ defects—while the self-trapped exciton (STE) is assigned to the band at 455 nm in which an oxygen atom is displaced to a neighboring oxygen site, thus trapping the electron and the hole in the corner-sharing [SiO_4_] tetrahedra that are present in the structure of cuprorivaite.

Figures S18–S25 of the ESI show the luminescence time-resolved measurements and the relative fits of the EB powder and of F2, F3, and F4 films at the emission wavelengths of 390 and 455 nm (λ_ex_ = 242 nm), while Table 1 summarizes the average lifetime values obtained.

It is interesting to note that the lifetimes for the EB powder are of the order of microseconds, as already measured by Binet at al. [26], which are attributed to forbidden triplet–singlet exciton recombination. An important finding is that lifetimes of the order of microseconds are also measured for the F2, F3, and F4 films (see Table 1), confirming that the emissive properties in the UV–visible range of EB are maintained after incorporation in the CMC films. The lifetime increases from the F2 (non-crosslinked) to F3/F4 (crosslinked) film. This effect can be related to the greater difficulty for the electron–hole pairs to meet in the crosslinked matrix, therefore causing the longer time decay of the exciton.

To ensure that the NIR emission of EB was maintained after incorporation in the CMC matrix, fluorescence spectroscopy in the NIR region was recorded. Upon excitation at 630 nm, which corresponds to the ^2^B_1g_ → ^2^E_g_ absorption band of Cu^2+^ [9], a broad emission band centered at 900 nm is observed in Figure 9. Moreover, two shoulder bands at 928 nm and 960 nm are also observed. As expected, the CMC film with 3% content of EB (F4) is the one that presents the highest intensity of emission, whereas the crosslinking does not seem to significantly affect it (F3 compared with F2).

In addition, the absolute luminescence quantum yield of the F2, F3, and F4 films at an excitation at 621 nm shows values of 10.9, 12.8, and 11.3%, with average lifetimes of 123.29 ± 0.59, 122.97 ± 0.69, and 122.10 ± 0.71 µs (Table 1 and Figures S26–S28 of the ESI), respectively. These values of quantum yields and lifetimes are of the same order of magnitude as the EB powder (11.0%; τ_av_ = 124.81 ± 1.64 µs, see Figure S29 of the ESI) [27], confirming that the unique NIR luminescence of the EB prevails after being incorporated in the CMC matrix. Furthermore, it is worth pointing out that these results represent a spring of inspiration for the incorporation of various luminescent complexes in sustainable carboxymethylcellulose matrixes [58,59,60,61].

## 3. Materials and Methods

### 3.1. Reagents

Sodium carboxymethylcellulose (CMC), average Mw ≈ 250,000 and DS = 1.2, was purchased from Sigma-Aldrich; Egyptian blue (EB) < 10 μm and citric acid 1-hydrate (CA) were purchased from Kremer Pigmente GmbH & Co (Wachtersbach, Germany) and from PanReac (Barcelona, Spain), respectively, and used as received.

### 3.2. Preparation of Films

Films of citric acid-crosslinked carboxymethylcellulose (CMC–CA) without and with the fluorophore EB were prepared using a solution casting method [37]. Several CMC aqueous solutions with percentages of 2.0%, 3.5%, 5.0%, 6.5%, and 10.0% (*w*/*v*) were prepared by stirring sodium carboxymethylcellulose in 100 mL of distilled water, at room temperature, until complete dissolution. Different weight percentages (ranging from 2.5 to 10 wt.%, *w*/*w* based on CMC weight) of citric acid (CA, crosslink agent) were added to the CMC solutions and stirred in for 30 min. The resulting CMC–CA solutions were cast into glass petri dishes and dried at 80 °C for 12 h in an oven to promote the crosslinking mechanism [20]. The dried films were peeled off and kept at room temperature in a desiccator for further characterization.

**Figure 10:**
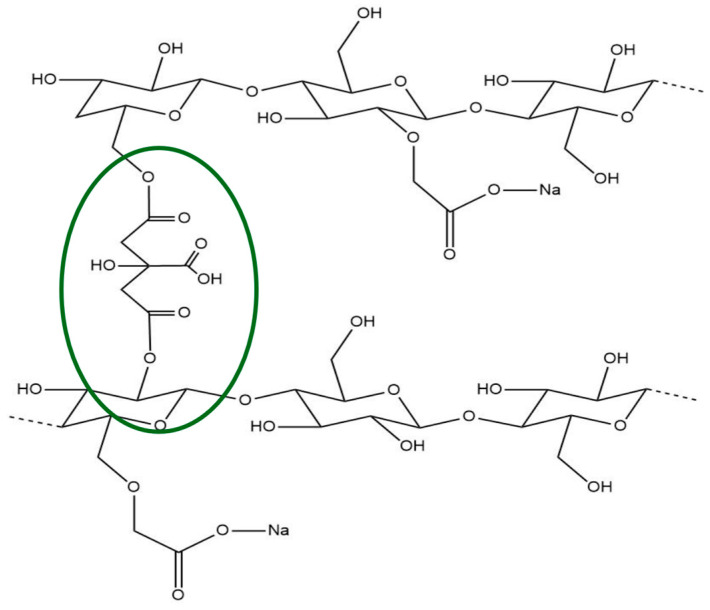
Carboxymethylcellulose (CMC) chains crosslinked with citric acid (CA) are circled in green (chemical structures drawn using ChemDraw Ultra 12.0 software).

Citric acid is a non-toxic crosslinking agent that modifies the chemical structure of CMC through esterification reactions between the hydroxyl groups present in the CMC polymer and the carboxylic groups of the acid, as illustrated in Figure 10.

This crosslinking process only occurs upon heating and provides films with higher thermochemical stability and structural stiffness.

From all the CMC aqueous solutions prepared, the solution with CMC 2% (*w*/*v*) yielded the thinnest and the most fragile films, while the solution with CMC 10% (*w*/*v*) could not even be completely solubilized. The combination of CMC 6.5% (*w*/*v*) with 10.0% (*w*/*v*) of CA was the one that provided the most resistant and flexible film and which ensured the adequate solubility of EB; therefore, it was the most suitable and the only one used from this point on. In fact, EB was added to 6.5% CMC aqueous solutions at concentrations of 0.5%, 1.5%, and 3.0% (*w*/*w* based on CMC weight) followed by manual stirring to ensure homogeneity. Crosslinked CMC–EB-CA films were obtained after drying at 80 °C for 12 h in an oven.

The samples were abbreviated from F0 to F4 according to their EB and CA, content as depicted in Table 2. Only these films were used for further characterization.

### 3.3. Characterization

An STA 300 thermal analysis system from Hitachi was used to perform both TGA and DSC techniques. All the analysis were carried out with a heating rate of 10 °C/min, under nitrogen atmosphere.

Attenuated total reflection (ATR) spectra were obtained using a Bruker Tensor 27 spectrophotometer (Billerica, MA, USA), equipped with a Golden Gate ATR horizontal cell. The established parameters were 256 scans between 4000–350 cm^−1^, with a resolution of 2 cm^−1^.

Coherent anti-Stokes Raman scattering (CARS) spectroscopy was applied by combining the emissions from two regenerative titanium–sapphire mode-locking laser oscillators with tunable picosecond pulse emissions (Tsunami model, Spectra Physics) which were coupled to a confocal microscope (model SP8, Leica). The λ_pump_ was set to 727 nm and the λ _Stokes_ was set to 750.46 nm, so the energy difference corresponded to a wavenumber of 430 cm^−1^, which was the most intense vibrational mode of EB and absent from the CMC matrix.

The pH measurements were performed at room temperature using a digital pH meter (model mPA210, MS TECNOPON, Piracicaba, Brazil), previously calibrated with standard buffer solutions of pH 4 and pH 7. The analyses were performed on CMC (6.5%) and CMC–EB (1.5%) solutions (precedents of the F0 and F2 films, respectively). The conductivity measurements were conducted at room temperature using a Nano Series ZS90 Zetasizer (Malvern Panalytical, Malvern, UK) with a DTS1070 polystyrene cuvette. All the measurements were performed in triplicate.

Zeta potential measurements on CMC (6.5%) and CMC–EB (1.5%) solutions were performed using a Nano Series Zetasizer (Malvern Panalytical, Malvern, UK). The samples, diluted with distilled water (10×), were put in a measurement cell and equilibrated at 25 °C for 5 min inside the instrument before data were collected (detection angle = 173°). Measurements were carried out in triplicate, and each individual measurement was an average of 12 runs. The instrument affords the zeta potential by analyzing the electrophoretic mobility of the particles by means of the Smoluchowski equation.

For the acquisition of AFM topography images, a Nanoscope Multimode III scanning probe microscope from Digital Instruments (Veeco Metrology Inc., Santa Barbara, CA, USA) was used, operating in tapping mode. Images were acquired at different scan areas (5 µm × 5 µm, 1 µm × 1 µm, and 500 nm × 500 nm), enabling the evaluation of the surface organization at multiple scales.

UV–Vis spectra were obtained with a Cintra 303 spectrophotometer from GBC (Absorbance mode, speed = 100 nm/min, step size = 0.520 nm, slit width = 5.0 nm).

The NIR luminescence of CMC–EB films was assessed through the Vis/Nir SENSE spectrometer with an integration time of 100 ms, a LS-LED light source of 630 nm, and a reflectance probe for solid samples. Time-resolved luminescent measurements were obtained by a FLS980 spectrofluorometer (Edinburgh Instruments Ltd.) using either the time-correlated single photon counting technique with an Edinburgh picosecond-pulsed LED (emitted wavelength 300 nm) or by multichannel scaling technique with a microsecond-pulsed Xenon flashlamp. Time-resolved luminescent curves were fitted using the following multiexponential function:

$$I\;\left(\lambda ,\;t\right)=\sum_{i=1}^{n}\alpha_{i}\left(\lambda \right)\;exp\left(\frac{-t}{\tau_{i}}\right)$$

where *n* is the number of exponentials and *α_i_* (*λ*) is the amplitude at wavelength *λ* and *τ_i_* is the lifetime of the component *i*. The quality of the fit was evaluated through the reduced *χ*^2^ value.

An average lifetime (τ_av_) was calculated using the following definition:

$$\tau_{\mathrm{AV}}=\frac{\sum_{i=1}^{n}\alpha_{i}\tau_{i}^{2}}{\sum_{i=1}^{n}\alpha_{i}\tau_{i}}$$

Absolute luminescent quantum yields (Φ) of the films were measured using a C11347 Quantaurus Hamamatsu Photonics K.K spectrometer (Hamamatsu, Japan), equipped with a detection system with a maximum wavelength of 960 nm and excitation wavelengths in the range of 280–800 nm. The estimated error in Φ is 0.1%. A description of the experimental setup and measurement method can be found in the article of K. Suzuki et al. [62]. Due to the lower limit of detection of the instrument (960 nm) compared to the emission spectrum of the CMC–EB films (which extends up to around 1020 nm) the calculated absolute luminescent quantum yield values are slightly underestimated. The diffuse reflectance spectrum (DRS) of the EB powder was determined using a UV-3600 Plus UV–Vis–NIR spectrophotometer (Shimadzu Italia S.r.l., Milan, Italy) equipped with an integrating sphere and using BaSO_4_ as a standard reference. Measurements were recorded between 200 and 1000 nm with 1 nm intervals and a spectral bandwidth of 20 nm.

## 4. Conclusions

The development of sustainable carboxymethylcellulose-based materials with multichannel luminescence capability is a research hotspot, and the incorporation of a luminophore emitting in the near infrared (NIR) into the carboxymethylcellulose (CMC) matrix would expand the number of applications of this technology. The broad excitation band in the visible part of the spectrum of Egyptian blue (EB) and its emission in the NIR region makes it a promising candidate. Additionally, CMC, in addition to providing a sustainable matrix, also presents blue fluorescence from the CTE mechanism due to the clustering of oxygen atoms and carboxyl groups (-COOH).

To the best of our knowledge, this is the first report of CMC-based materials incorporating Egyptian blue (EB) as a luminophore exhibiting emissions in both the visible and NIR regions. The success of the crosslinking mechanism through an esterification-based mechanism using citric acid (CA) as a crosslinking agent as well as the success of the incorporation of the EB into the CMC matrix were confirmed by attenuated total reflection (ATR) and ultraviolet–visible (UV–Vis) spectroscopy. Zeta potential (ZP) analysis revealed strong electrostatic interactions and improved colloidal stability between CMC and EB.

The inclusion of EB retained its characteristic NIR emission with a peak λ_em_ max = ~950 nm, achieving quantum yield values between 11.2% and 12.8%, while also ensuring stable dispersion within the CMC matrix, as verified by CARS imaging and zeta potential measurements. Furthermore, the CMC films displayed the typical clustering-triggered emission (CTE) in the blue region at 430 nm, with a slight increase in luminescence quantum yield (Φ) from 5.8% to 6.1% after crosslinking with citric acid.

This work demonstrates the potential of CMC–EB-CA films for several applications, such as in bioimaging, sensing, and security marking.

## Figures and Tables

**Table 1:** Average lifetime (τ_av_) and absolute luminescent quantum yield (Φ) values of the Egyptian blue powder and of the F2, F3, and F4 films. The estimated error in Φ is 0.1%.

Sample	τ_av_ λ_ex_ = 242 nm λ_em_ = 390 nm	τ_av_ λ_ex_ = 242 nm λ_em_ = 455 nm	τ_av_ λ_ex_ = 621 nm λ_em_ = 840 nm	Φ (%) λ_ex_ = 620 nm
EB powder	109.54 ± 0.38 µs	128.84 ± 0.49 µs	124.81 ± 1.64 µs	11.0
F2	55.90 ± 1.68 µs	115.62 ± 4.36 µs	123.29 ± 0.59 µs	10.9
F3	99.50 ± 2.25 µs	124.05 ± 3.70 µs	122.97 ± 0.69 µs	12.8
F4	149.05 ± 4.13 µs	150.19 ± 6.10 µs	122.10 ± 0.71 µs	11.3

**Table 2:** CMC–EB-CA films prepared and used for further characterization. All samples are based on the host matrix CMC 6.5% (*w*/*v*), described above.

CMC 6.5-EB-CA	Egyptian Blue/%(*w*/*w*)	Citric Acid/% (*w*/*w*)
F0	-	-
F1	-	10
F2	1.5	-
F3	1.5	10
F4	3.0	10

## Data Availability

Normalized emission spectra, excited state decay measurements, and related fits have been included as part of the Supplementary Information.

## Supplementary Materials

The following supporting information can be downloaded at: https://www.mdpi.com/article/10.3390/molecules30112359/s1, Average lifetimes (τ_av_) and absolute luminescent quantum yield (Φ) of the F0 and F1 films are presented in Table S1; Comparison between the ATR spectra of citric acid, non-crosslinked CMC film (F0), and CMC–CA-crosslinked (F1) films is shown in Figure S1 of the ESI; AFM topography images of F1 film acquired at different scan areas: (A) 5 µm × 5 µm, (B) 1 µm × 1 µm and (C) 500 nm × 500 nm in Figure S2 of the ESI; UV–Vis spectra of non-crosslinked CMC film (F0) and CMC–CA-crosslinked (F1) films are presented in Figure S3 of the ESI; pictures of non-crosslinked CMC (F0) and citric acid-crosslinked CMC (F1) films under a 365 nm UV light lamp are reported in Figure S4 of the ESI; Jablonski diagrams of F0 and F1 are reported in Figures S5 and S6 of the ESI; excited state decay measurements and related fits are reported in Figures S7–S10, S14–S16 and S18–S29 of the ESI; normalized emission spectra are reported in Figures S17 of the ESI; Jablonski diagrams of EB and F2–F4 are shown in Figures S11–S13 of the ESI.

## Abbreviations

The following abbreviations are used in this manuscript:

AFM	Atomic force microscopy
ATR	Attenuated total reflection
CA	Citric acid
CARS	Coherent anti-Stokes Raman scattering
CMC-CA	Citric acid-crosslinked carboxymethylcellulose
CMC	Carboxymethylcellulose
CTE	Clustering-triggered emission
DSC	Differential scanning calorimetry
NIR	Near-infrared
PL	Photoluminescence
TGA	Thermogravimetric analysis
ZP	Zeta potential
