# Supplementary material for: Egyptian Blue into Carboxymetylcellulose: New Dual-Emissive Solid-State Luminescent Films
Source: Molecules. 2025 May 28;30(11):2359. doi: 10.3390/molecules30112359 (PMC12156393; doi:10.3390/molecules30112359)

Supporting Information for

Egyptian Blue into Carboxymetylcellulose: New Dual Emissive Solid-State Luminescent Films

Mariana Coimbra [1,#,‡], Francesco Fagnani [2,‡], Gisele Peres [3], Paulo Ribeiro-Claro [1], Juan Carlos Otero [4], Daniele Marinotto [5], Dominique Roberto [2,*], Mariela Nolasco [1,*]

[1] CICECO – Instituto de Materiais de Aveiro, Departamento de Química, Universidade de Aveiro, P-3810-193 Aveiro, Portugal.
[2] Dipartimento di Chimica, Università degli Studi di Milano, UdR-INSTM, Via C. Golgi 19, 20133 Milan, Italy.
[3] Federal University of Fronteira Sul, 85301-970, Laranjeiras do Sul - PR, Brazil.
[4] Department of Physical Chemistry, Faculty of Sciences, University of Málaga, E-29071 Málaga, Spain
[5] Istituto di Scienze e Tecnologie Chimiche (SCITEC) "Giulio Natta", Consiglio Nazionale delle Ricerche (CNR), via C. Golgi 19, I-20133 Milan, Italy.
‡ These authors have contributed equally to this work.
# Present filiation, Center for Cooperative Research in Biomaterials (CIC biomaGUNE), Basque Research and Technology Alliance (BRTA), Paseo de Miramon 194, Donostia-San Sebastiaán 20014, Spain

* Correspondence: dominique.roberto@unimi.it; mnolasco@ua.pt

Figure S1. Comparison between ATR spectra of citric acid (CA), non-crosslinked CMC film (F0) and CMC-CA crosslinked (F1) films.

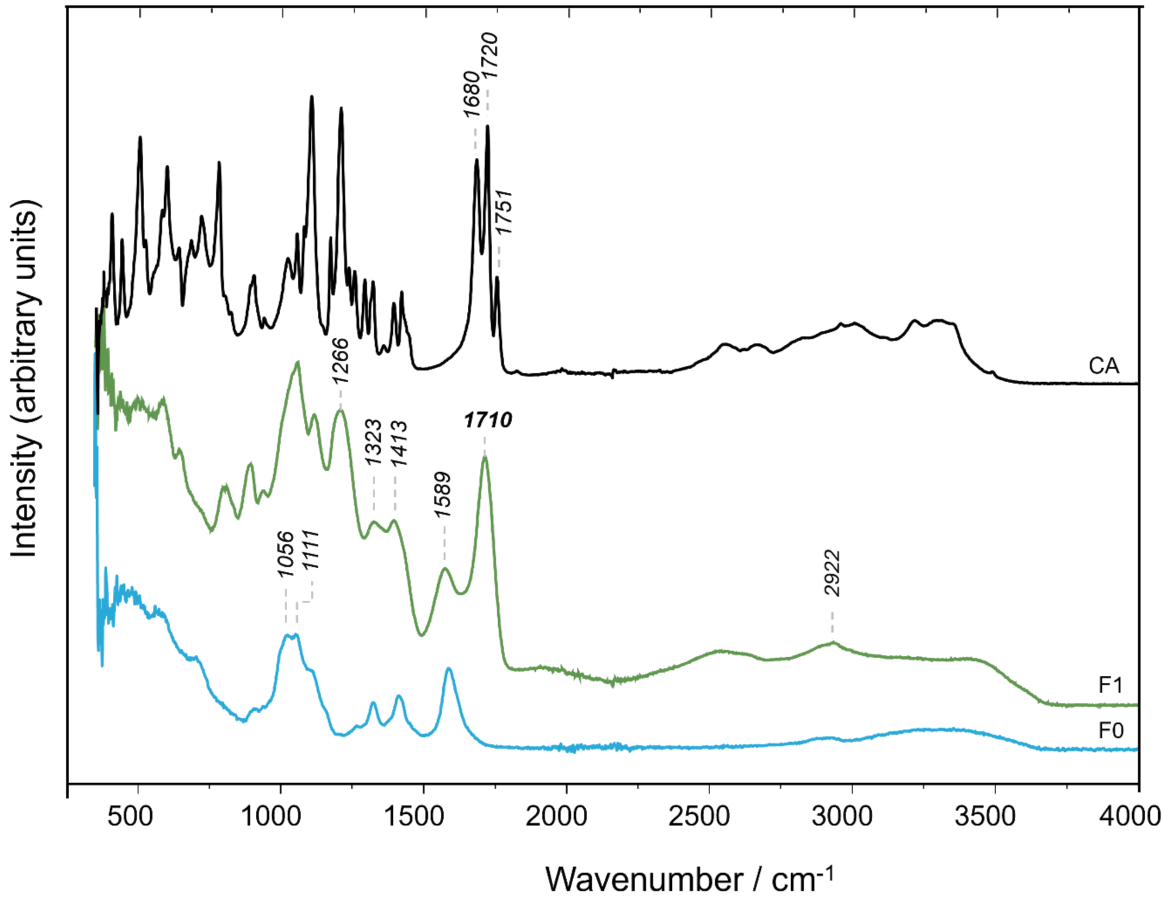

Figure S2. AFM topography images of F1 film acquired at different scan areas: (A) 5 µm × 5 µm,(B)1 µm × 1 µm and (C) 500 nm × 500 nm.

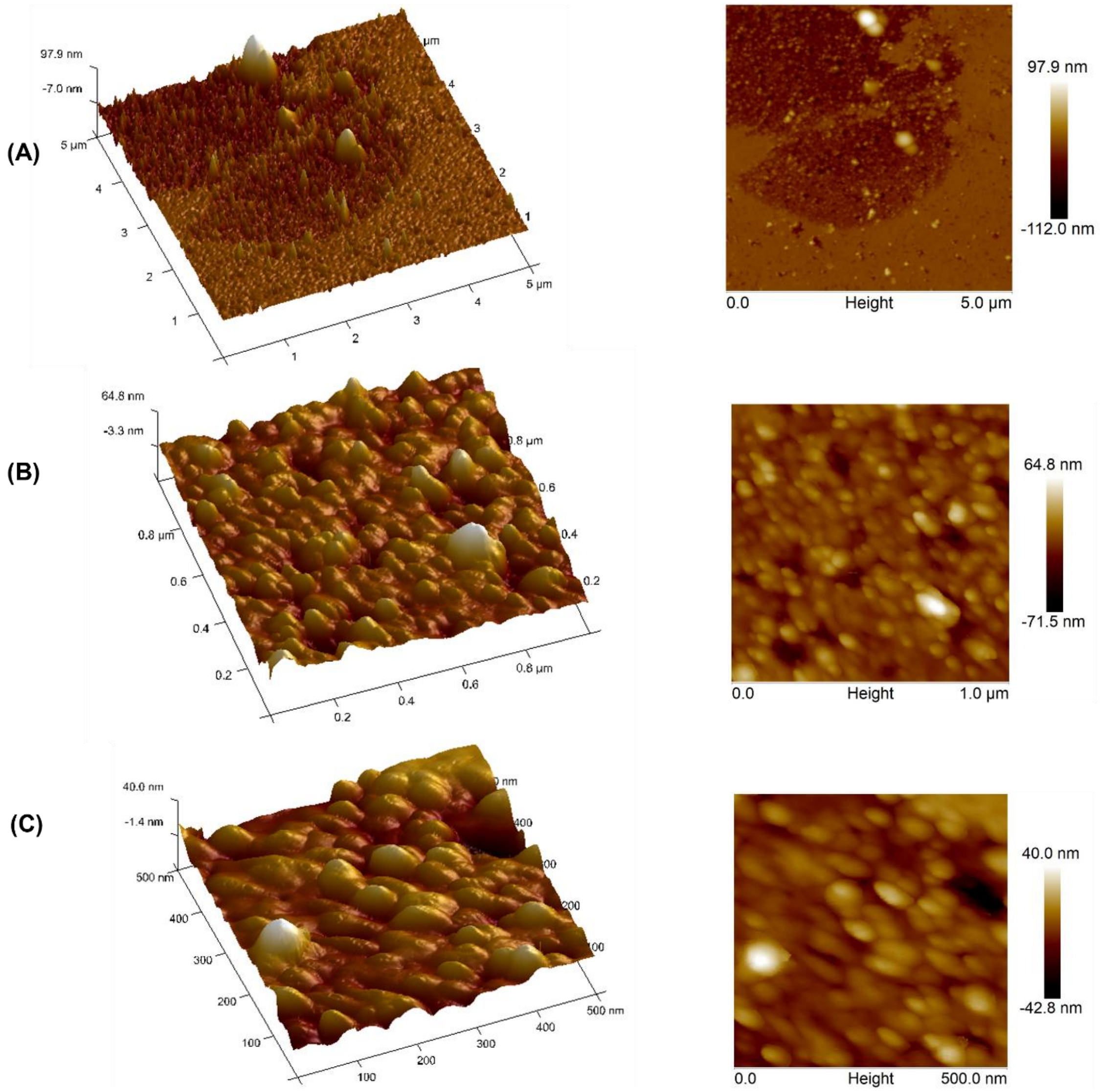

Figure S3. UV-Vis spectra of non-crosslinked CMC film (F0) and CMC-CA crosslinked (F1) films.

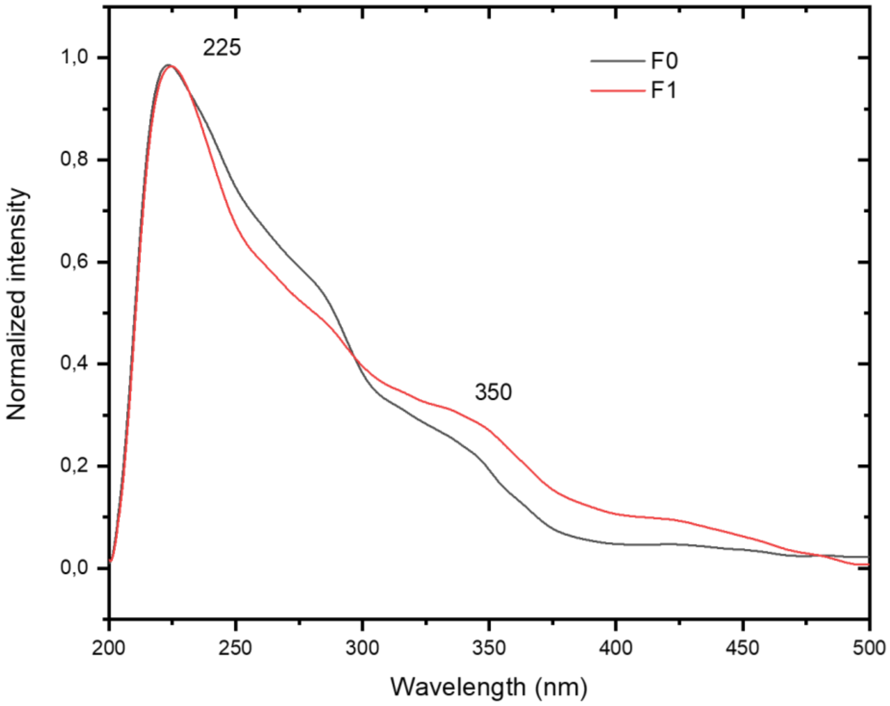

Figure S4. (A) Non-crosslinked CMC (F0) and (B) citric acid crosslinked CMC (F1) films under a 365 nm UV light lamp.

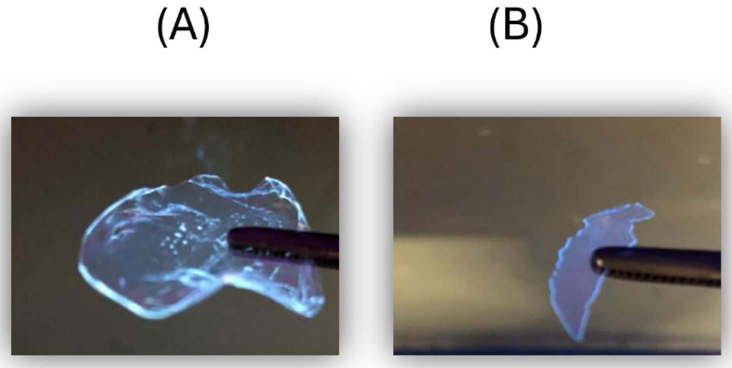

Figure S5. Jablonski diagram of F0 non-crosslinked matrix.

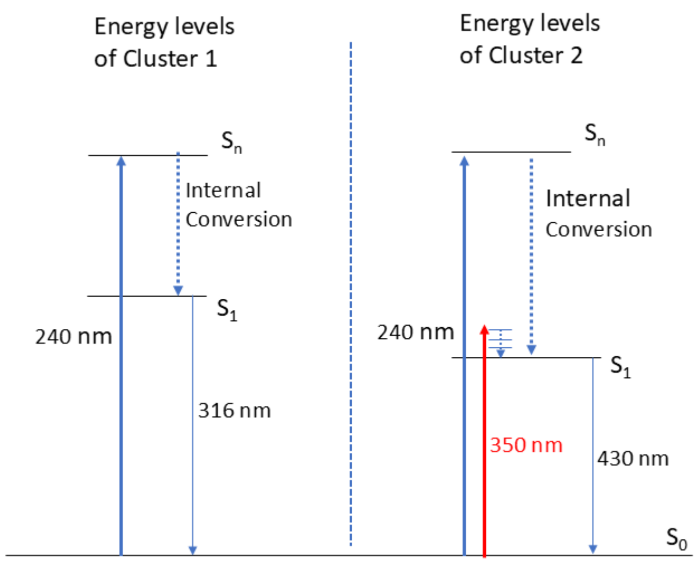

Figure S6. Jablonski diagram of F1 crosslinked matrix.

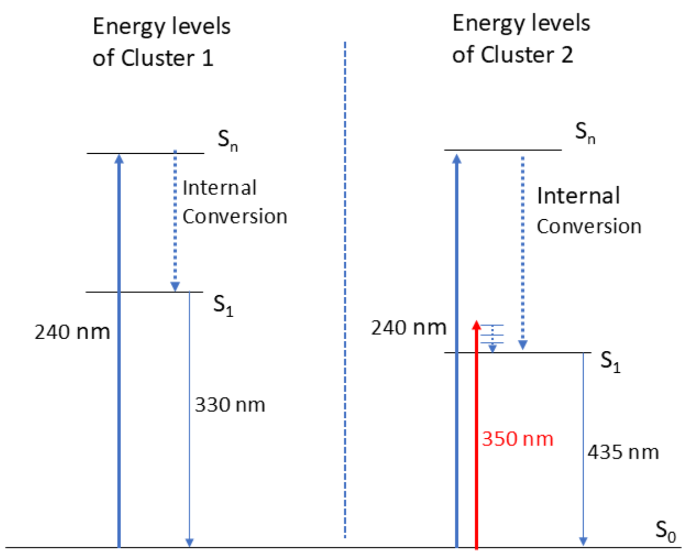

Figure S7. Excited state decay measurement and relative fitting of the F0 film. Excitation wavelength 300 nm, emission wavelength 316 nm.

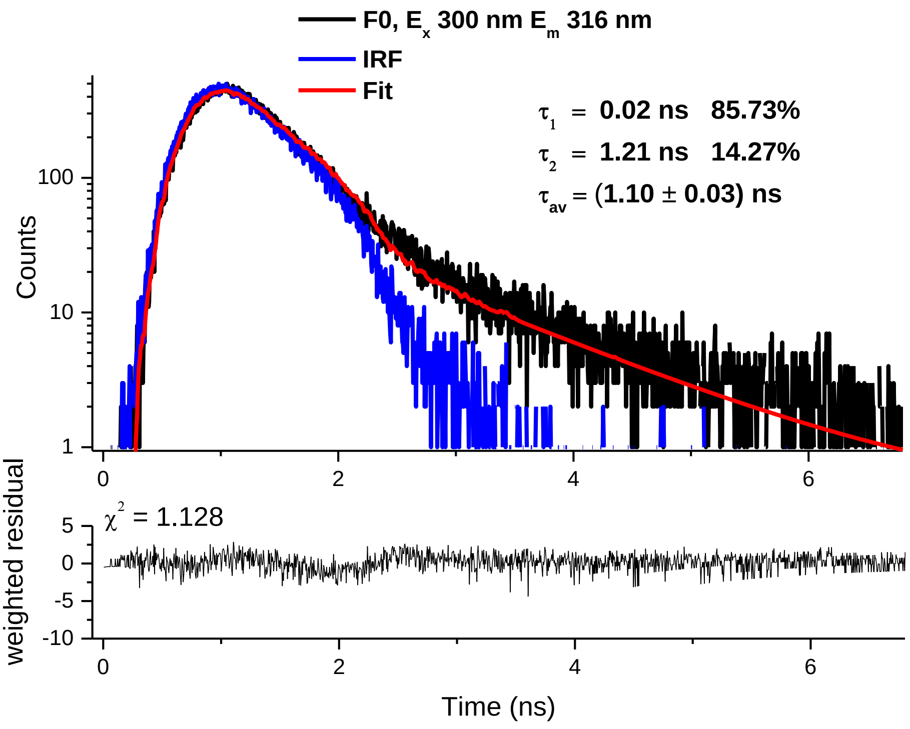

Figure S8. Excited state decay measurement and relative fitting of the F1 film. Excitation wavelength 300 nm, emission wavelength 330 nm.

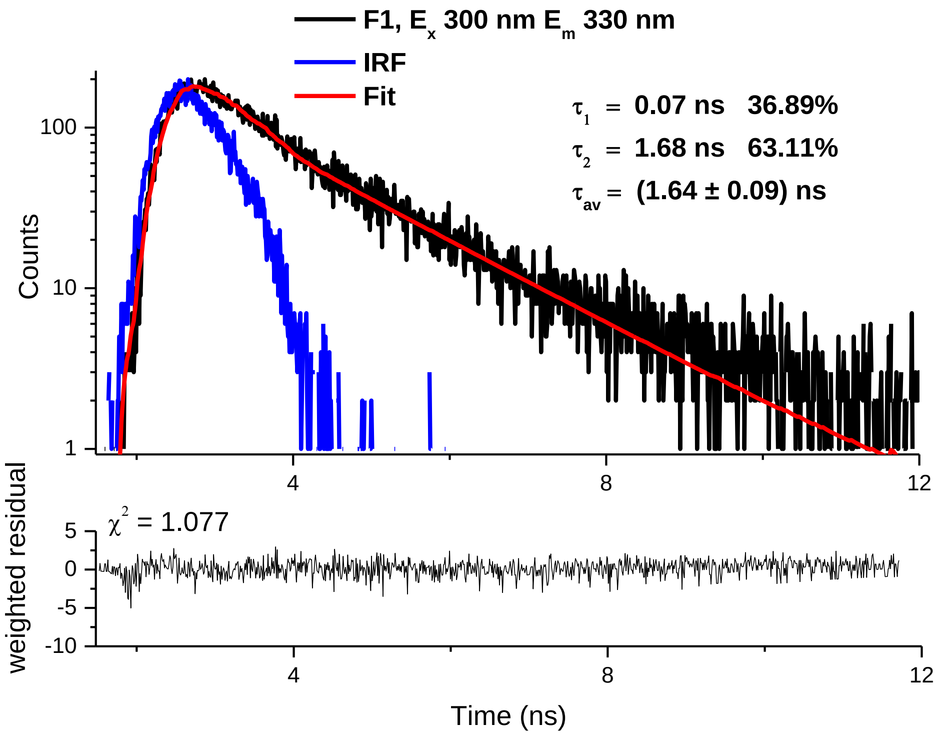

Figure S9. Excited state decay measurement and relative fitting of the F0 film. Excitation wavelength 300 nm, emission wavelength 430 nm.

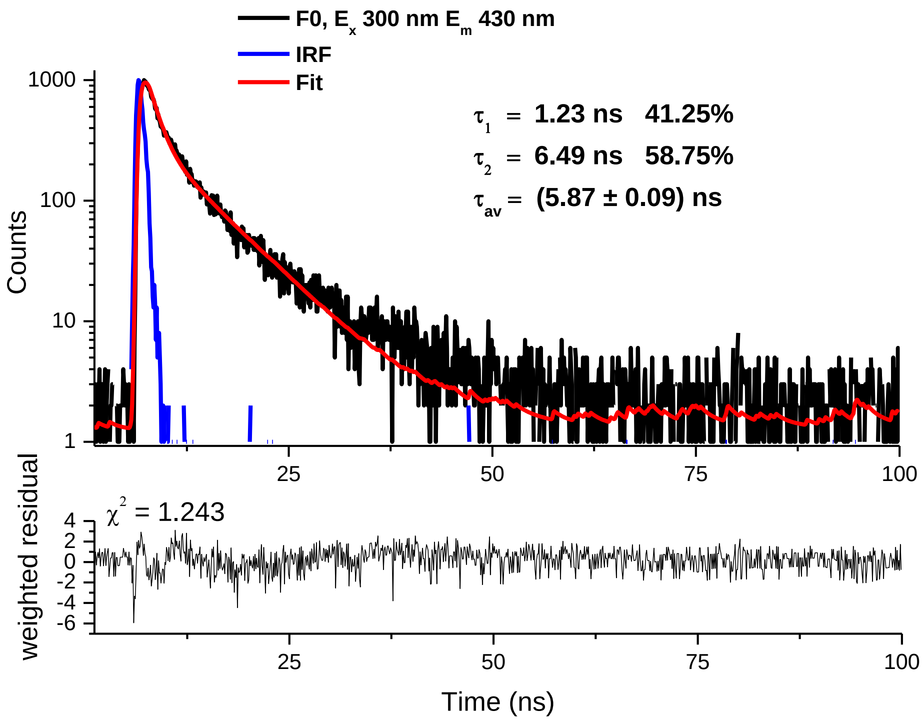

Figure S10. Excited state decay measurement and relative fitting of the F1 film. Excitation wavelength 300 nm, emission wavelength 435 nm.

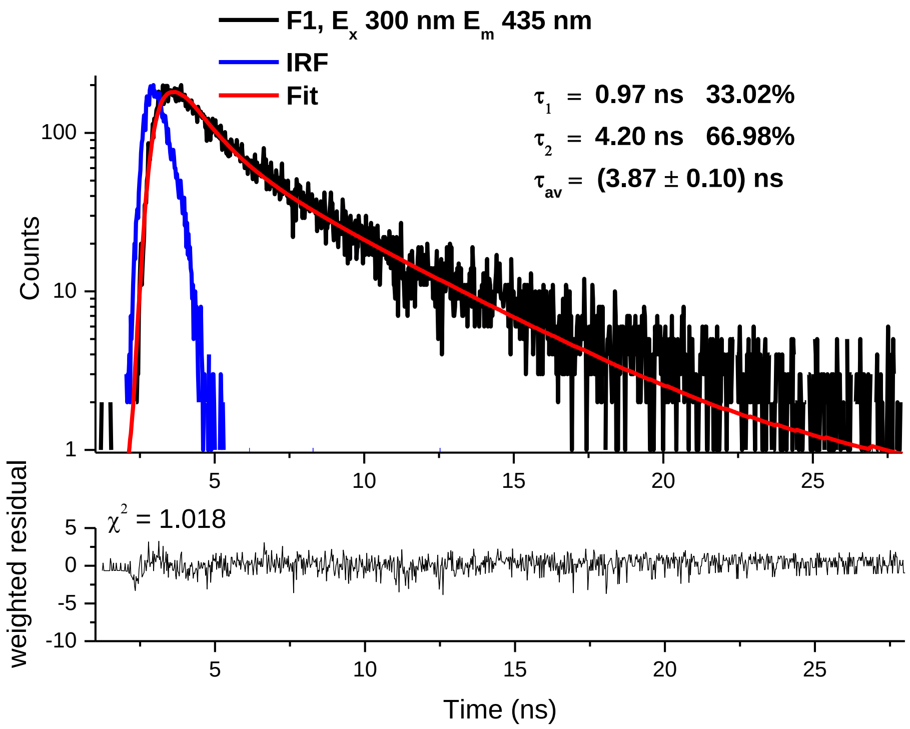

Figure S11. Jablonski diagram of cuprorivaite powders, adapted from *J. Phys. Chem. C* **2021**, 125, 25189-25196; doi: 10.1021/acs.jpcc.1c06060

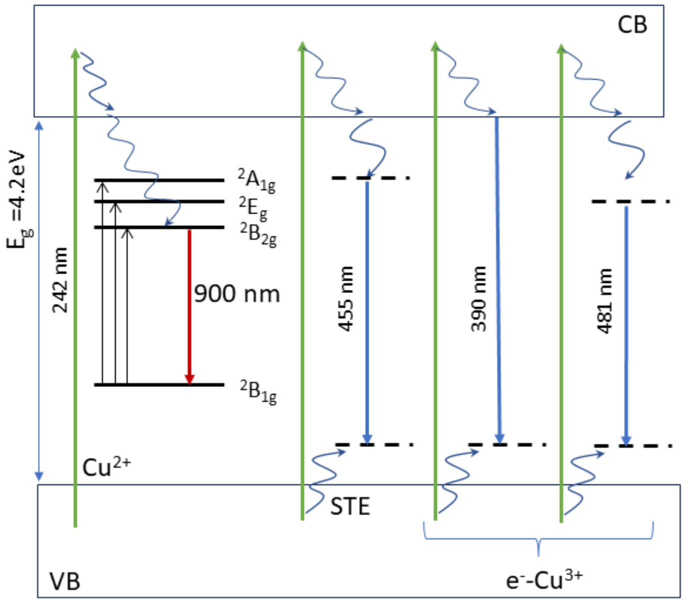

Figure S12. Jablonski diagram of F2 with non-crosslinked matrix.

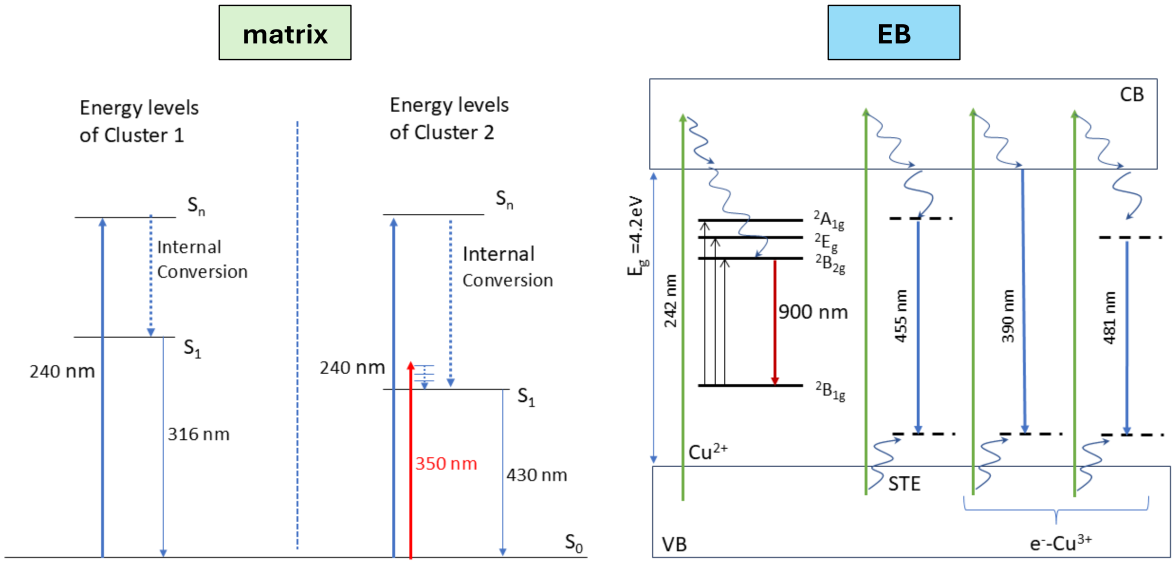

Figure S13. Jablonski diagram of F3 and F4 with crosslinked matrix.

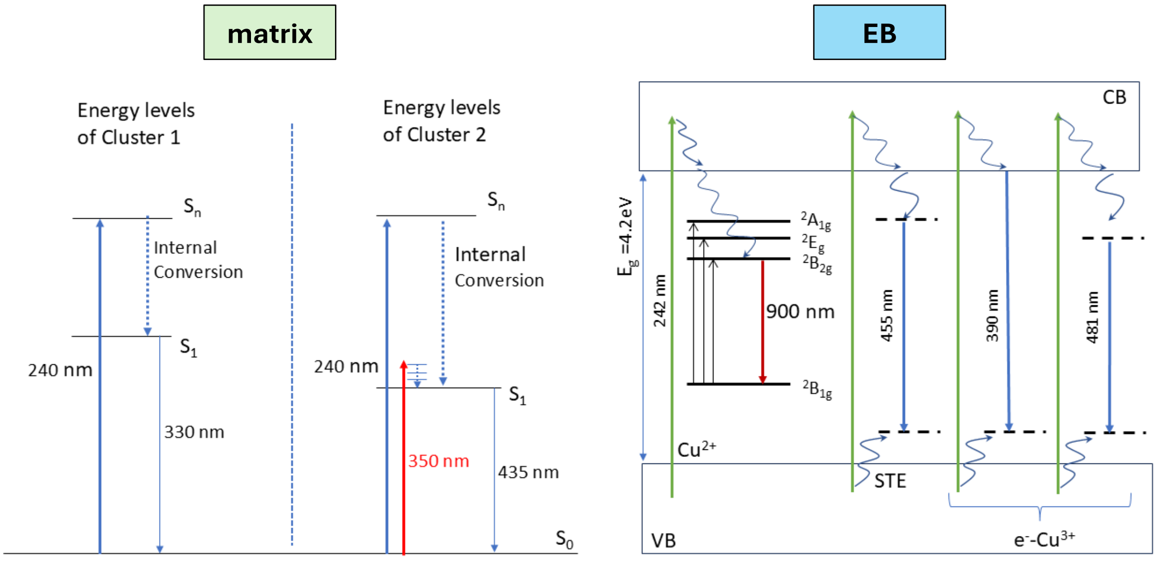

Table S1. Average lifetimes (τ_{av}) and absolute luminescent quantum yield (Φ) of the F0 and F1 films. The estimated error in Φ is 0.1%.

Sample	τ_{av} λ_{ex} =300 nmà λ_{em} = 430 nm	Φ (%) λ_{ex} =350 nm
**F0**	5.87 ± 0.09 ns	5.8
**F1**	3.87 ± 0.10 ns	6.1
**F2**	4.79 ± 0.06 ns	1.3
**F3**	4.43 ± 0.06 ns	2.0
**F4**	4.59 ± 0.06 ns	1.4

Figure S14. Excited state decay measurement and relative fitting of the F2 film. Excitation wavelength 300 nm, emission wavelength 430 nm.

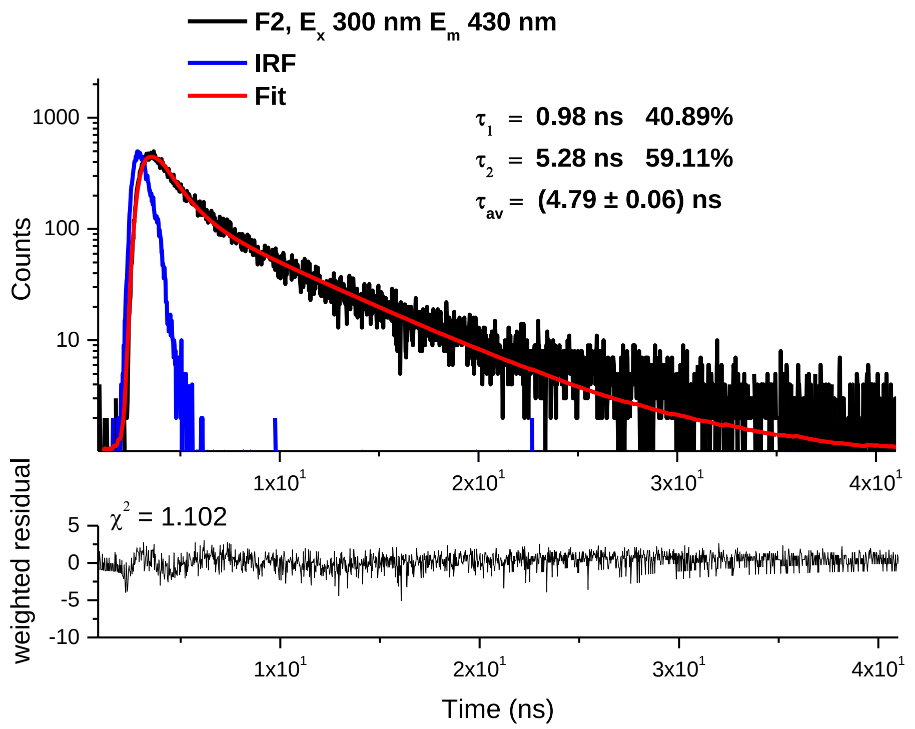

Figure S15. Excited state decay measurement and relative fitting of the F3 film. Excitation wavelength 300 nm, emission wavelength 430 nm.

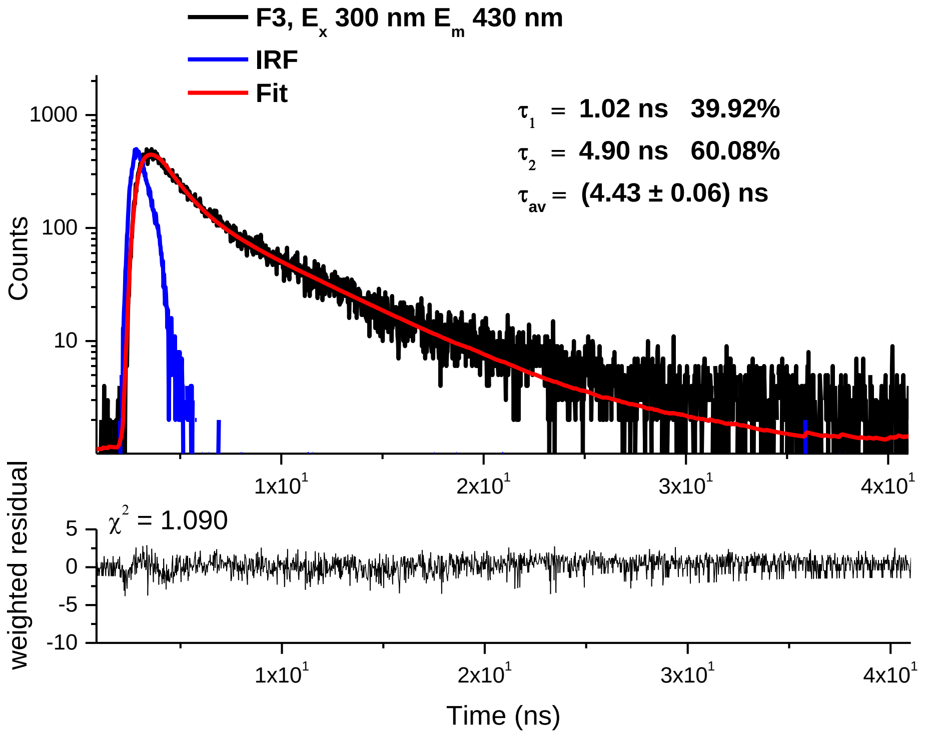

Figure S16. Excited state decay measurement and relative fitting of the F4 film. Excitation wavelength 300 nm, emission wavelength 430 nm.

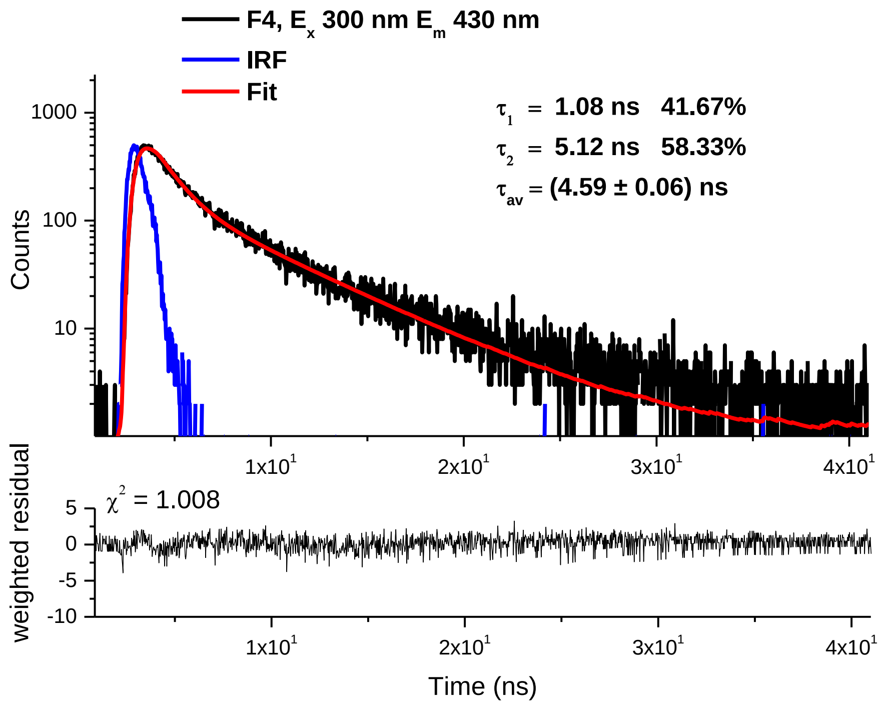

Figure S17. Normalized emission spectra of Egyptian blue (EB) powder and of F2, F3 and F4 films, excitation wavelength 242 nm

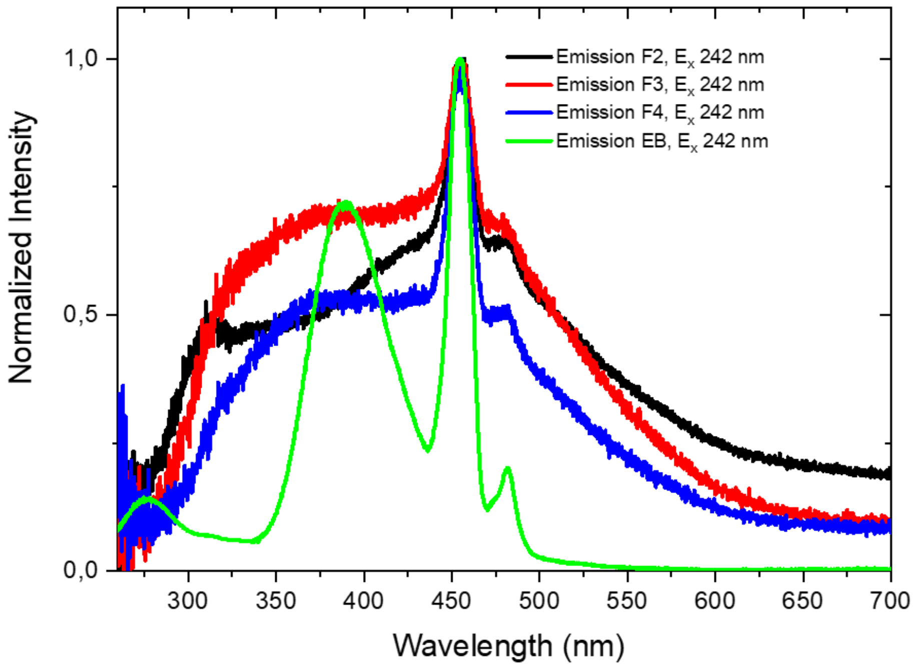

Figure S18. Excited state decay measurement and relative fitting of the EB powder. Excitation wavelength 242 nm, emission wavelength 390 nm.

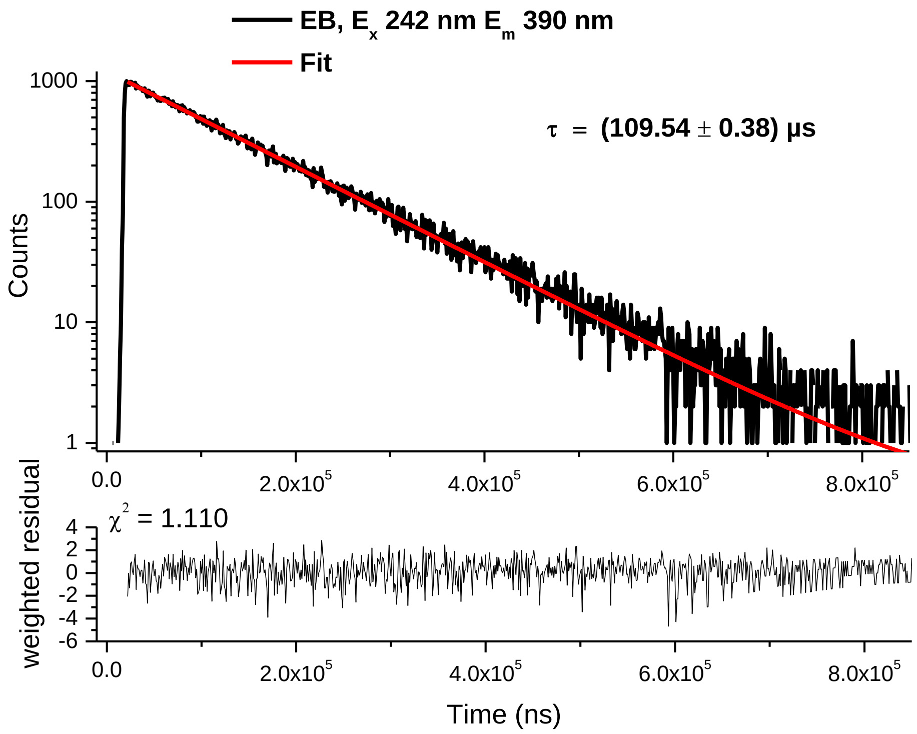

Figure S19. Excited state decay measurement and relative fitting of the EB powder. Excitation wavelength 242 nm, emission wavelength 455 nm

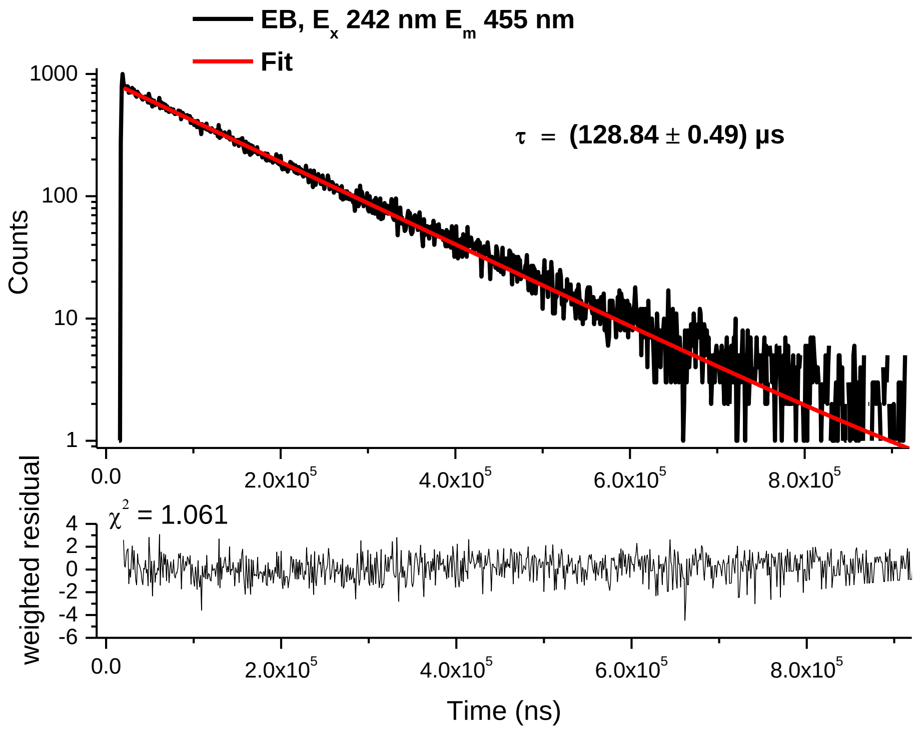

Figure S20. Excited state decay measurement and relative fitting of the F2 film. Excitation wavelength 242 nm, emission wavelength 390 nm.

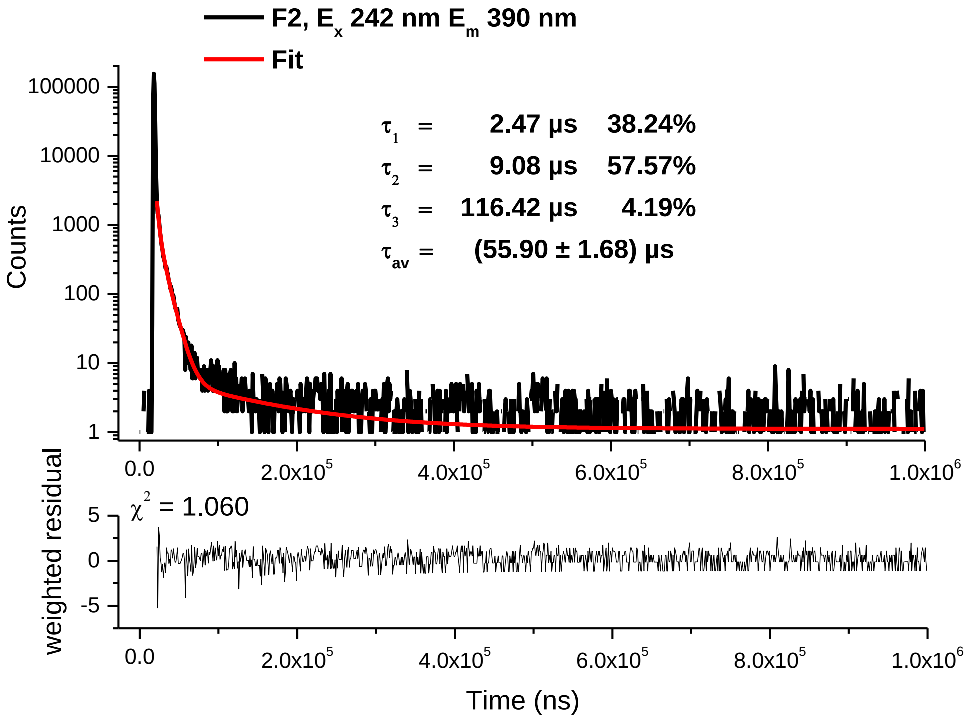

Figure S21. Excited state decay measurement and relative fitting of the F2 film. Excitation wavelength 242 nm, emission wavelength 455 nm.

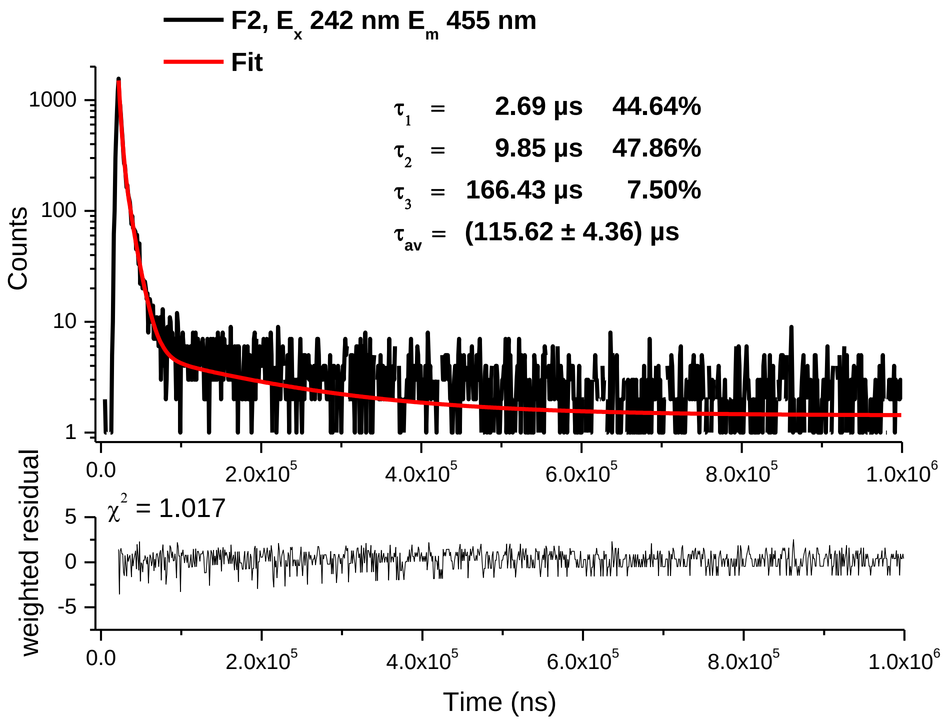

Figure S22. Excited state decay measurement and relative fitting of the F3 film. Excitation wavelength 242 nm, emission wavelength 390 nm.

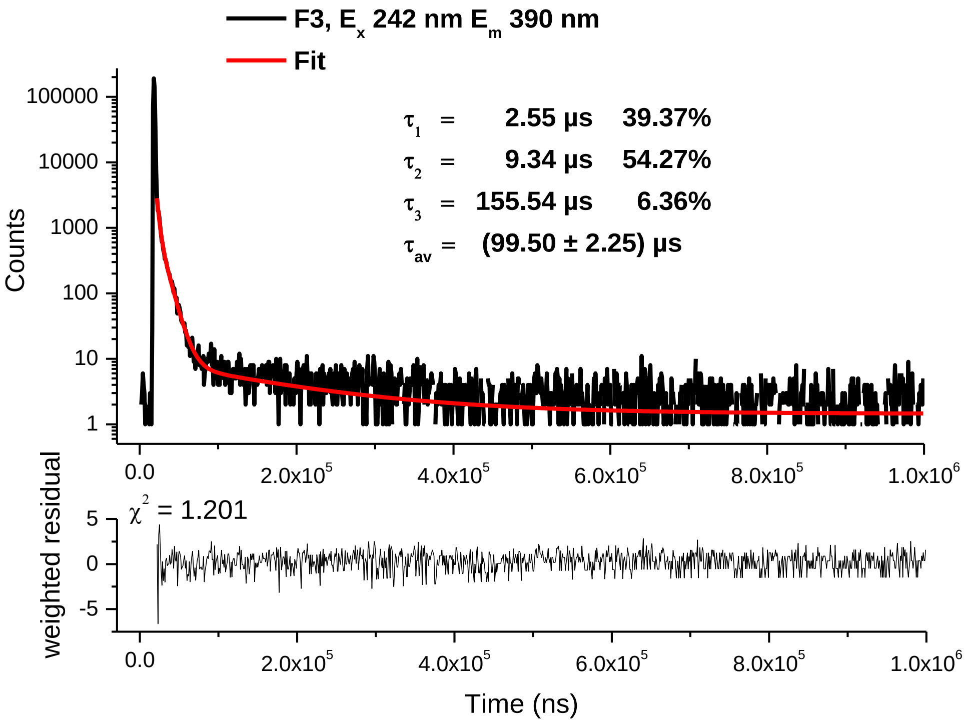

Figure S23. Excited state decay measurement and relative fitting of the F3 film. Excitation wavelength 242 nm, emission wavelength 455 nm.

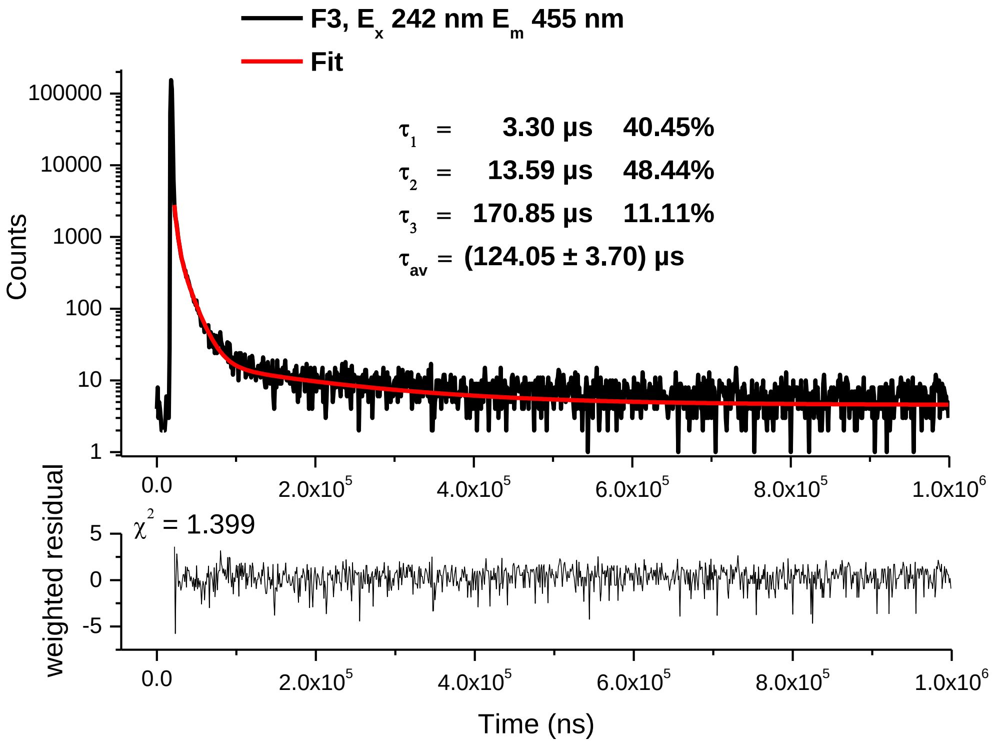

Figure S24. Excited state decay measurement and relative fitting of the F4 film. Excitation wavelength 242 nm, emission wavelength 390 nm.

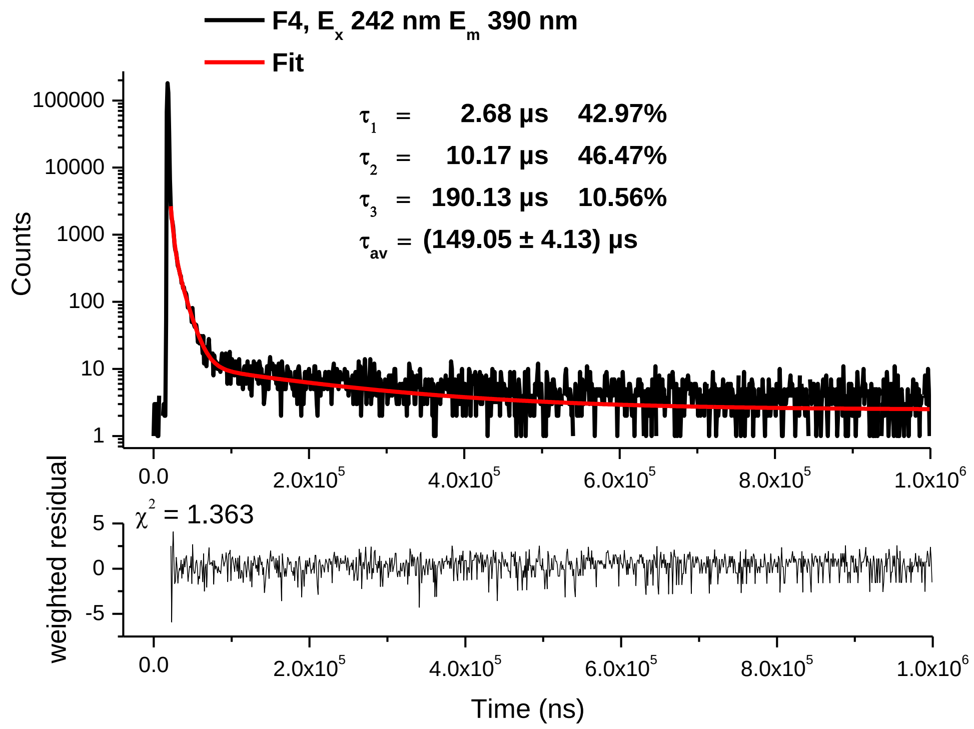

Figure S25. Excited state decay measurement and relative fitting of the F4 film. Excitation wavelength 242 nm, emission wavelength 455 nm.

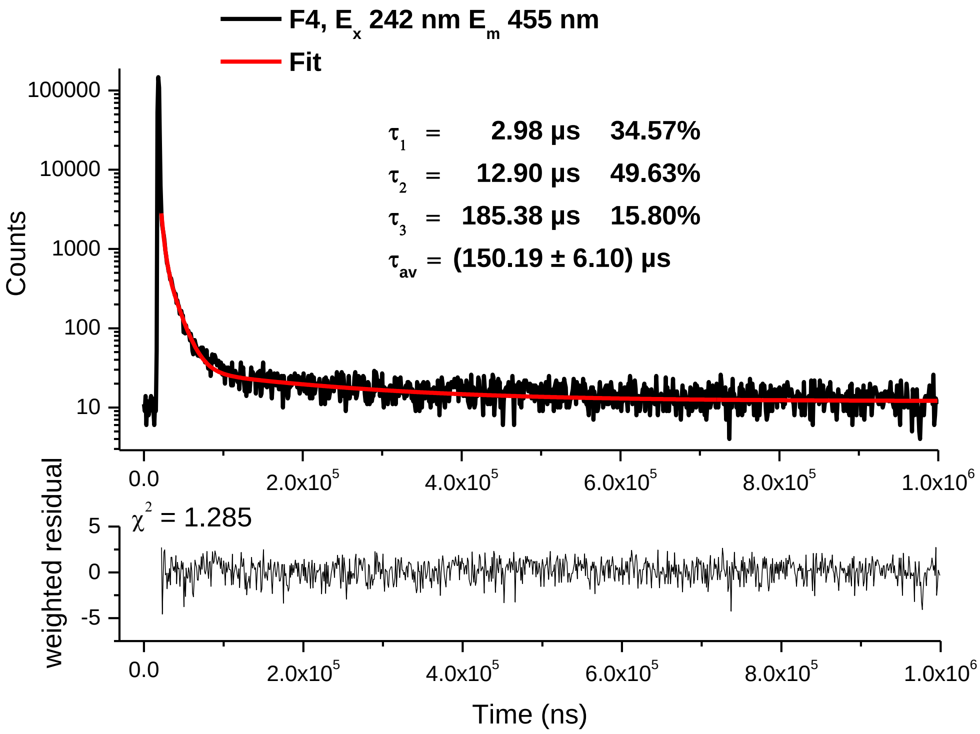

Figure S26. Excited state decay measurement and relative fitting of the F2 film. Excitation wavelength 621 nm, emission wavelength 840 nm.

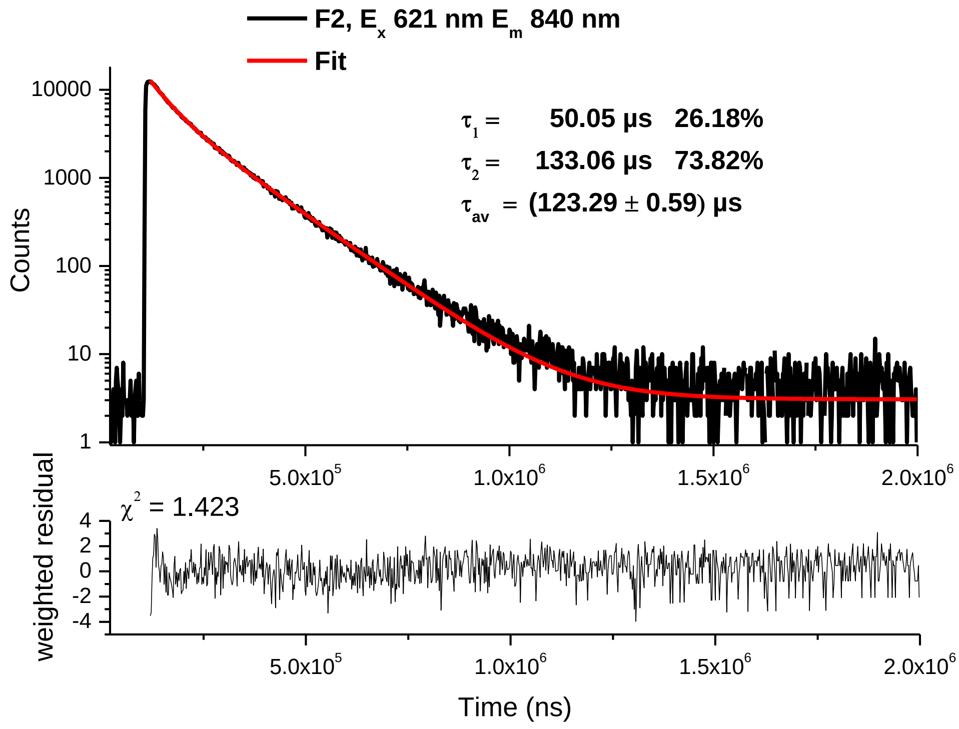

Figure S27. Excited state decay measurement and relative fitting of the F3 film. Excitation wavelength 621 nm, emission wavelength 840 nm.

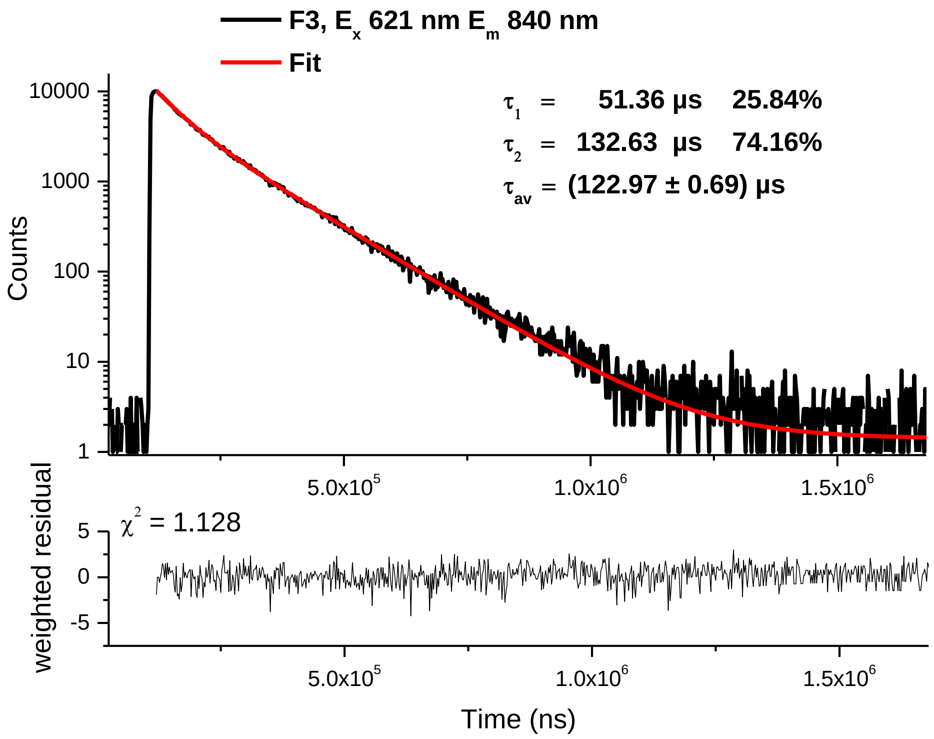

Figure S28. Excited state decay measurement and relative fitting of the F4 film. Excitation wavelength 621 nm, emission wavelength 840 nm

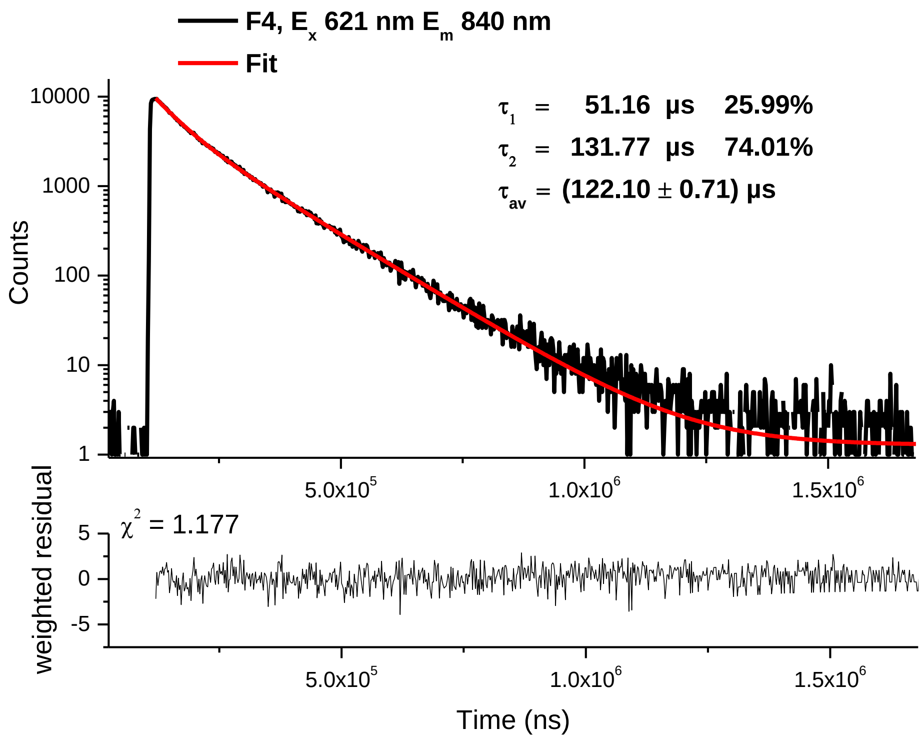

Figure S29. Excited state decay measurement and relative fitting of the EB powder. Excitation wavelength 621 nm, emission wavelength 840 nm.

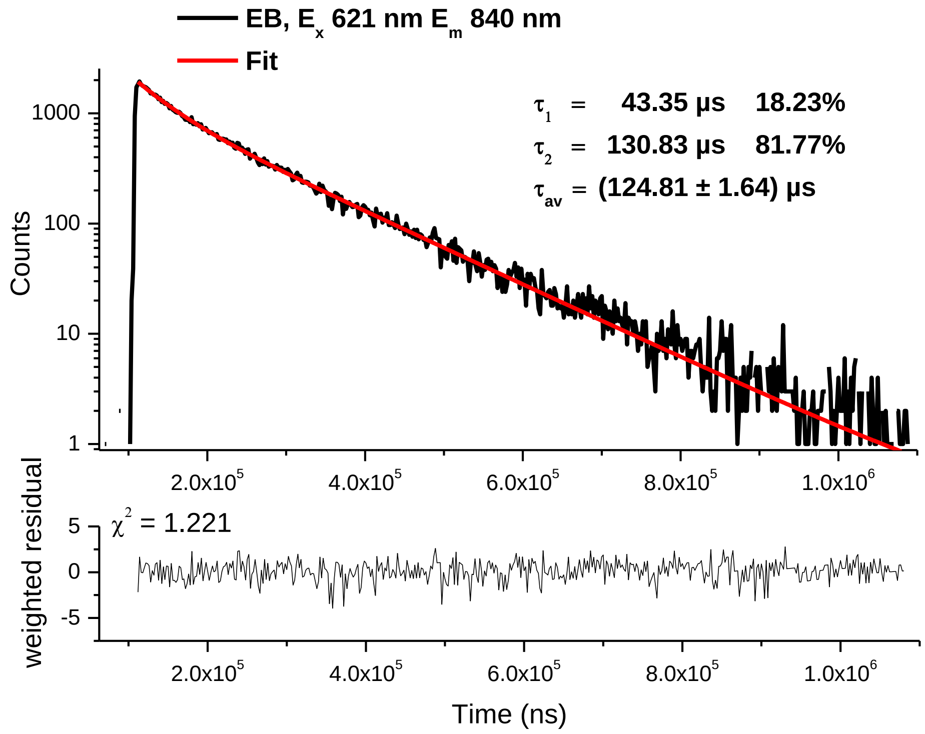

Supplement: Supplementary file 1 [file molecules-30-02359-s001.zip › molecules-3624115-supplementary.pdf]
